# QiShenYiQi Pill Ameliorates Cardiac Fibrosis After Pressure Overload-Induced Cardiac Hypertrophy by Regulating FHL2 and the Macrophage RP S19/TGF-β1 Signaling Pathway

**DOI:** 10.3389/fphar.2022.918335

**Published:** 2022-07-13

**Authors:** Gulinigaer Anwaier, Ting-Ting Xie, Chun-Shui Pan, An-Qing Li, Li Yan, Di Wang, Fan-Kai Chen, Ding-Zhou Weng, Kai Sun, Xin Chang, Jing-Yu Fan, Jing-Yan Han, Jian Liu

**Affiliations:** ^1^ Department of Integration of Chinese and Western Medicine, School of Basic Medical Sciences, Peking University, Beijing, China; ^2^ Tasly Microcirculation Research Center, Peking University Health Science Center, Beijing, China; ^3^ Academy of Integration of Chinese and Western Medicine, Peking University Health Science Center, Beijing, China; ^4^ Key Laboratory of Microcirculation, State Administration of Traditional Chinese Medicine of the People’s Republic of China, Beijing, China; ^5^ Key Laboratory of Stasis and Phlegm, State Administration of Traditional Chinese Medicine of the People’s Republic of China, Beijing, China; ^6^ State Key Laboratory of Core Technology in Innovative Chinese Medicine, Tianjin, China; ^7^ Beijing Microvascular Institute of Integration of Chinese and Western Medicine, Beijing, China

**Keywords:** cardiac fibrosis, pressure overload, FHL2, RP S19, TGF-β1, SMADs, heart failure, QiShenYiQi pill

## Abstract

**Purpose:** Heart failure (HF) is a leading cause of morbidity and mortality worldwide, and it is characterized by cardiac hypertrophy and fibrosis. However, effective treatments are not available to block cardiac fibrosis after cardiac hypertrophy. The QiShenYiQi pill (QSYQ) is an effective treatment for chronic HF. However, the underlying mechanism remains unclear.

**Methods:** In the present study, a pressure overload-induced cardiac hypertrophy model was established in rats by inducing ascending aortic stenosis for 4 weeks. QSYQ was administered for 6 weeks, and its effects on cardiac fibrosis, myocardial apoptosis, RP S19 release, macrophage polarization, TGF-β1 production, and TGF-β1/Smad signaling were analyzed. *In vitro* studies using H9C2, Raw264.7, and RDF cell models were performed to confirm the *in vivo* study findings and evaluate the contribution to the observed effects of the main ingredients of QSYQ, namely, astragaloside IV, notoginsenoside R1, 3,4-dihydroxyl-phenyl lactic acid, and *Dalbergia odorifera* T. C. Chen oil. The role of four-and-a-half LIM domains protein 2 (FHL2) in cardiac fibrosis and QSYQ’s effects were assessed by small interfering RNAs (siRNAs).

**Results:** QSYQ ameliorated cardiac fibrosis after pressure overload-induced cardiac hypertrophy and attenuated cardiomyocyte apoptosis, low FHL2 expression, and TGF-β1 release by the injured myocardium. QSYQ also inhibited the following: release of RP S19 from the injured myocardium, activation of C5a receptors in monocytes, polarization of macrophages, and release of TGF-β1. Moreover, QSYQ downregulated TGF-βR-II expression induced by TGF-β1 in fibroblasts and inhibited Smad protein activation and collagen release and deposition.

**Conclusion:** The results showed that QSYQ inhibited myocardial fibrosis after pressure overload, which was mediated by RP S19-TGF-β1 signaling and decreased FHL2, thus providing support for QSYQ as a promising therapy for blocking myocardial fibrosis.

## Introduction

Heart failure (HF) is a common outcome of various cardiovascular diseases and has poor prognosis (Yancy et al., 2013). Despite progress in the treatment of HF, the prevalence and hospitalization rates are still increasing (Bacmeister et al., 2019). In response to mechanical stress or hemodynamic factors, such as pressure overload, the cardiac ventricular muscle adapts by undergoing concentric hypertrophy. However, when pressure overload is sustained, the muscle becomes maladaptive and gradually progresses to cardiac fibrosis, which eventually leads to cardiac dysfunction and HF (Vonk Noordegraaf et al., 2019). However, the pathological processes and mechanisms underlying the progression of overload-induced cardiac hypertrophy to irreversible fibrosis are not fully understood.

Mounting evidence has established the role of cardiac myocyte apoptosis in the regulation of the progression of cardiovascular disease to heart failure (Chiong et al., 2011). However, the mechanism by which apoptotic cardiac myocytes respond to sustained pressure overload to accelerate cardiac fibrosis has not been clarified. Our previous study demonstrated the involvement of ribosomal protein S19 (RP S19)-mediated transforming growth factor β1 (TGF-β1) signaling in I/R-induced myocardial fibrosis (Zheng et al., 2019). RP S19 is mainly released from apoptotic cells and promotes monocyte infiltration by binding to the monocyte complement activation fragment 5a receptor (C5aR). However, whether similar signaling occurs in sustained pressure overload injured myocardial cells remains unclear.

The four-and-a-half LIM-domain protein 2 (FHL2) is the second member of the four-and-a-half LIM domain protein family, and it is most abundant in the heart tissue (Chan et al., 1998). Evidence shows that FHL2 protein levels are lower in patients with hypertrophic cardiomyopathy (HCM), and FHL2 overexpression protects phenylephrine- or endothelin-1-induced hypertrophy in cardiac myocytes (Friedrich et al., 2014). Furthermore, a recent study showed that FHL2 interacts with TGF-β1 and inhibits its expression (Dahan et al., 2017) and is involved in kidney fibrosis (Duan et al., 2020), liver fibrosis (Huss et al., 2013), and lung fibrosis (Alnajar et al., 2013). However, whether FHL2 participates in cardiac fibrosis in response to chronic pressure overload has not been reported.

QiShenYiQi Pill^®^ (QSYQ) is a compound Chinese medicine approved by the China State Food and Drug Administration in 2003 for the treatment of cardiac dysfunction (Wang et al., 2011), and it is composed of *Astragalus mongholicus* Bunge (Huangqi, Fabaceae family), *Panax notoginseng* (Burkill) F. H. Chen (Sanqi, Araliaceae), *Salvia miltiorrhiza* Bunge (Danshen, mint family, Lamiaceae), and *Dalbergia odorifera* T. C. Chen (Jiangxiang, Dalbergia family, Leguminosae) (Zhang et al., 2012). The major active ingredients of QSYQ are astragaloside IV (ASIV, Huangqi), notoginsenoside R1 (R1, Sanqi), 3,4-dihydroxy-phenyl lactic acid (DLA, Danshen), and *Dalbergia odorifera* T. C. Chen oil (DO, Jiangxiang). Clinical data show that QSYQ partially reverses left ventricular hypertrophy in hypertensive patients (Daoyong et al., 2012), improves heart function in patients with chronic HF (Zheng-bin et al., 2015), and increases exercise tolerance in patients with ischemic heart failure (IHF) (Mao et al., 2020). In addition, research has confirmed that QSYQ improves the ejection fraction and fractional shortening score of animals with HF induced by transverse aortic constriction (Ruan et al., 2018) and acute myocardial infarction (Wang et al., 2015). Our previous study showed that QSYQ posttreatment alleviated pressure overload-induced cardiac hypertrophy (Chen et al., 2015). However, previous studies have not focused on whether QSYQ and its main ingredients ameliorate cardiac fibrosis secondary to cardiac hypertrophy or identified the underlying mechanism.

In the present study, an ascending aortic stenosis (AAS) rat model was used to investigate the effects and mechanisms of QSYQ and its main ingredients ASIV, R1, DLA, and DO on cardiac fibrosis caused by pressure overload. The results of the *in vitro* study using cardiomyocytes, monocytes, and fibroblasts provide further insights into the mechanisms underlying the effects of QSYQ.

## Material and Methods

### Reagents and Antibodies

QSYQ (batch number: 20030139) was obtained from Tasly Pharmaceutical Co. Ltd. (Tianjin, China). Trimetazidine hydrochloride tablets (TMZ, batch number: H20055465) were obtained from Servier Pharmaceutical Co., Ltd. (Tianjin, China) for the *in vivo* study. Both QSYQ and TMZ were dissolved in saline (Beijing Chemical Works, Beijing, China) to generate solutions with concentrations of 0.16 g/ml (Chen et al., 2015) and 2 mg/ml (Ma et al., 2019), respectively. ASIV, DLA, and R1 (purity ≥99.9%) were obtained from Feng Shan Jian Medicine Research Co. Ltd. (Kunming, Yunnan, China). DO (batch number: 20121101) was obtained from Tasly Pharmaceutical Co. Ltd. (Tianjin, China). Trimetazidine dihydrochloride (TMZ) and angiotensin II (Ang II) used in the *in vitro* studies were purchased from Selleck (Radnor, Pennsylvania, United States).

Wheat germ agglutinin (WGA, Alexa Fluor^®^ 594 conjugate) and rhodamine-phalloidin were purchased from Invitrogen (Carlsbad, CA, United States). Hematoxylin and Eosin (HE) was purchased from Zhongshan Goldenbridge Biotechnology Co., Ltd. (Beijing, China). Antibodies against TGF-β1, p-Smad3, Smad3, Smad4, Smad6, Smad7, matrix metalloproteinases 9 (MMP9), MMP2, tissue inhibitor of metalloproteinases 1 (TIMP1), TIMP2, RP S19, and FHL2 were purchased from Abcam (Cambridge, United Kingdom); antibodies against CD68, CD163, and CD80 were purchased from Santa Cruz Biotechnology (Santa Cruz, Calif, United States); antibodies against TGFβR II, Bax, Bcl-2, cleaved caspase-3, caspase-3, cleaved caspase-3, caspase-3, and GAPDH were obtained from Cell Signaling Technology (Beverly, MA, United States); and antibodies against C5aR, collagen I, and collagen III were obtained from Proteintech (Proteintech Group, Inc., Rosemont, United States). Enzyme-linked immunosorbent assay (ELISA) kits for Adenosine triphosphate (ATP), Adenosine diphosphate (ADP), Adenosine monophosphate (AMP), lactate dehydrogenase (LDH), malondialdehyde (MDA), TGF-β1, monocyte chemoattractant protein-1 (MCP-1), RP S19, and N-terminal pro-brain natriuretic peptide (NT-ProBNP) were purchased from Beijing Andihuatai Technology Co. Ltd. (Beijing, China).

### Animals

Male Sprague–Dawley rats (95–100 g) were purchased from the Animal Center of the Peking University Health Science Center (Beijing, certificate no. SCXK 2011–0012). Animals were housed at a temperature of 20 ± 2°C and a humidity of 40 ± 5% with 12/12-h light/dark cycles and fed standard rat chow and water. Animal care complied with the institutional guidelines of the Peking University Animal Research Committee, and the experimental protocols were approved by the Committee on the Ethics of Animal Experiments of Peking University Health Science Center (LA2020308) and consistent with the Guide for the Care and Use of Laboratory Animals published by the National Institute of Health (NIH). All efforts were made to minimize animal suffering and the number of sacrificed animals based on the rules of replacement, refinement, or reduction.

### AAS Surgical Protocols

Rats were fasted for 12 h before the experiment while allowed free access to water. The animals were anesthetized with 2% pentobarbital sodium (60 mg/kg) via intraperitoneal injection (Zheng et al., 2019). After tracheotomy, the animals were ventilated using a positive pressure respirator (ALC-V8, Shanghai, China). The chest was opened, and ascending aortic stenosis was implemented by placing a silver clip (0.9-mm inside diameter) on the ascending aorta. Sham-operated animals underwent the same procedure but without the clip (Chen et al., 2015).

### Drug Administration and Experimental Groups

After 4 weeks of AAS challenge, the animals were examined by echocardiography, and the rats with a left ventricular wall 20% thicker than that in the sham group were identified as successful in modeling (Supplementary Figures S8A–C) and randomly assigned to the model groups (AAS4W and AAS10W) and drug treatment groups. The animals in the sham group were randomly divided into the Sham4W, Sham10W, Sham10W + TMZ, and Sham10W + QSYQ groups. The animals in the drug treatment groups were administered QSYQ (0.8 g/kg/d) (Chen et al., 2015) or TMZ (10 mg/kg/d) (Mahfoudh Boussaid et al., 2016; Ma et al., 2019) for the next 6 weeks by gavage. The dose of 0.8 g/kg/d is 5 times equivalent to the clinical dose of QSYQ for adults (Shang et al., 2013). The animals in the Sham4W and AAS4W groups were sacrificed to assess the parameters of interest after the echocardiographic analysis. The animals in the Sham10W and AAS10W groups were examined using echocardiography at week 10 and then sacrificed for parameter assessment.

### Histological Analysis

The harvested hearts were cut horizontally, and the upper one-third was fixed in 4% paraformaldehyde solution for 48 h and processed for paraffin sections (7 μm) using an Automatic Paraffin slicer (Leica 2M2255, Leica, Mannheim, Germany). The sections were stained with HE, Masson’s trichrome, or Sirius red. The sections were observed under a microscope (BX512DP70; Olympus, Tokyo, Japan), and four fields were randomly selected for evaluation. Collagen accumulation in the interstitial and perivascular areas was analyzed using ImageJ 1.53c software (Version:2.1.0, National Institutes of Health, Maryland, United States) to assess the degree of cardiac fibrosis.

### Immunohistochemical Staining

Following dewaxing in xylene and rehydration in ethanol, antigen retrieval was performed in 0.01 mol/L sodium citrate buffer (pH 6.0) in a microwave. Endogenous peroxidase inhibition and blocking were performed. The paraffin sections were then incubated overnight with antibodies against CD68 (1:100), CD80 (1:100), CD163 (1:50), TGF-β1 (1:100), or RP S19 (1:100) after blocking with bovine serum albumin. Specific binding was detected by incubation with an HRP-conjugated secondary antibody, and positive staining was visualized with a DAB substrate kit. PBS was used instead of the primary antibody as a control. Images were captured using a digital camera connected to a microscope (BX512DP70; Olympus, Japan). The number of CD68/CD80/CD163-positive cells and the mean optical density of TGF-β1 and RP S19 were determined using ImageJ 1.53c software (Version: 2.1.0, National Institutes of Health, Maryland, United States).

### TUNEL Staining

Cardiomyocyte and H9C2 apoptosis were determined using an *in situ* Cell Death Detection kit, Fluorescein (Roche Diagnostics GmbH, Mannheim, Germany) according to the manufacturer’s protocol. Paraffin sections were dewaxed, hydrated, and permeabilized with protease K before incubation with 50 μL TUNEL reaction mixture (enzyme solution: label solution = 1:9) for 2 h in a 37°C incubator. H9C2 cells were seeded on 8-well glass slides (Thermo Fisher Scientific Inc. Rochester, NY, United States), and after the corresponding treatment, the cells were fixed with methanol and incubated with the TUNEL reaction mixture for 2 h in a 37°C incubator. Thereafter, paraffin sections and cells were counterstained with Hoechst 33342 for nuclear staining. Samples that were added only to the label solution served as negative controls. Images were acquired using a laser scanning confocal microscope (TCS SP8 STED 3X; Leica, Mannheim, Germany).

### Myocardial Ultrastructure

Fresh myocardial tissues (1 mm^3^) were excised from the left ventricle of rats 4 or 10 weeks after AAS. The tissues were fixed with 3% glutaraldehyde and post-fixed with 1% osmium tetroxide. The specimens were processed into ultrathin sections and stained with uranium acetate and lead citrate, and the ultrastructure of the myocardial tissue was examined using a transmission electron microscope (JEM 1400 plus, JEOL, Tokyo, Japan).

### Cell Culture and Treatment

H9C2 cells, a rat cardiac myoblast cell line (ATCC, Manassas, VA, United States), RDFs, a rat fibroblast cell line (ATCC, Manassas, VA, United States), and Raw264.7 cells, a mouse monocyte/macrophage cell line (ATCC, Manassas, VA, United States), were used for the *in vitro* studies and cultured in Dulbecco’s modified Eagle’s medium (Invitrogen, Carlsbad, CA, United States) containing 4 mM L-glutamine, 4.5 g/L glucose, and 10% fetal bovine serum (Invitrogen, Carlsbad, CA, United States) at 37°C in a humidified incubator with 95% air and 5% CO_2_. All cells were used in passages 5–10. The cells were seeded, grown to 70–80% confluence, and starved in a serum-free medium for 24 h before treatment. H9C2 cells were treated with 1 μM Ang II (Selleckchem, Philadelphia, United States) for 24 h for the *in vitro* studies (Wang et al., 2019a; Wang et al., 2019b). QSYQ and its main ingredients ASIV, R1, DLA, and DO were added 30 min before Ang II stimulation at 100 μg/ml, 0.56 μg/ml, 0.51 μg/ml, 3.63 μg/ml, and 3.43 μg/ml, respectively (Chen et al., 2015). The dosage of QSYQ (100 μg/ml) was selected based on our preliminary experiments (Supplementary Figures S9A–D). The dosages of the main ingredients *in vitro* were calculated based on the dosage ratio of the effective components in QSYQ and the results of our previous studies (Chen et al., 2015). TMZ (10 μM) was used as the positive control. Raw264.7 cells were treated with 100 ng/ml RP S19 (Proteintech, Rosemount, United States) for 24 h to simulate monocyte activation (Zheng et al., 2019), and QSYQ and its main ingredients were added 30 min before RP S19 stimulation. TMZ was used as the positive control. RDF cells were treated with 10 ng/ml TGF-β1 (Proteintech, Rosemount, United States) for 24 h to simulate myofibroblast activation (Yang et al., 2019), and QSYQ and its main components were added 30 min before RP S19 stimulation. TMZ was used as the positive control.

### Cardiomyocyte Cross-Section Area and Immunofluorescence Staining

The cardiomyocyte cross-sectional area was determined in four randomly selected fields at ×40 magnification of the objective in each section from the left ventricle, which was stained with WGA (5.0 μg/ml) and observed with a laser scanning confocal microscope (TCS SP8 STED 3X, Leica, Mannheim, Germany). The average cross-sectional areas of 7–10 cells in each section were calculated using ImageJ 1.53c software (Version:2.1.0, National Institutes of Health, Maryland, United States). F-actin was labeled with rhodamine-phalloidin (1:50).

Cells were fixed using 4% paraformaldehyde in PBS for 20 min at room temperature and permeabilized with 0.3% Triton X-100 at 37°C for 30 min. After blocking with 1% BSA and 22.52 mg/ml glycine in PBST (PBS+ 0.1% Tween 20) at room temperature for 30 min, monocytes and fibroblasts were incubated overnight at 4°C with anti-C5aR (1:50) plus anti-RP S19 (1:100) and anti-TGFβR I (1:100) plus anti-TGFβR II (1:100) antibodies, respectively, in 1% BSA in PBST. H9C2 cells were incubated overnight at 4°C with anti-FHL2 (1:200) and anti-TGF-β1 (1:100). After three washes in PBS, cells were incubated for 2 h at 37°C with DyLight-488/549-labeled secondary antibodies. The cells were counterstained with Hoechst 33342 for nuclei staining. Immunostaining without primary antibodies served as the control. Images were acquired using a laser scanning confocal microscope (TCS SP8 STED 3X; Leica, Mannheim, Germany).

### ELISA Assay

ELISA was performed using a specific kit to determine the levels of ATP, ADP, AMP, LDH, MDA, TGF-β1, RP S19, and MCP-1 in the myocardium and myocyte supernatants (Andygene, Beijing, China) and collagen I, collagen III, and TGF-β1 in fibroblast supernatants (Andygene, Beijing, China) using a microplate reader (Multiskan MK3, Thermo, Waltham, MA, United States). Each test was performed according to the manufacturer’s instructions.

### Western Blotting Assay

Approximately 50 mg of myocardium tissue was harvested from each animal (n = 4), quickly frozen in liquid nitrogen, and stored at −80°C for a maximum of 1 week before use. The total protein content was extracted using a protein extraction kit (Andygen, Texas, United States) and mixed with a 5× electrophoresis sample buffer. After electrophoresis, the separated proteins were transferred onto polyvinylidene difluoride membranes. After blocking with 5% nonfat dry milk, the membrane with target proteins was incubated overnight at 4°C with antibodies against RP S19, TGF-β1, TGFβR II, Smad3, p-Smad3, Smad4, Smad6, Smad7, MMP2, MMP9, TIMP1, TIMP2, collagen 1, collagen 3, cleaved caspase-3, caspase-3, cleaved caspase-9, caspase-9, cathepsin B, or GAPDH. After washing, the membrane was incubated with the secondary antibody for 1 hour at room temperature, and immunoreactive bands were detected using an enhanced chemiluminescence system. The protein signal was quantified by scanning densitometry and further evaluated using a bioimage analysis system (Quantity One image analyzer software, Bio-Rad, Richmond, CA, United States).

### Small Interfering RNA Transfection

FHL2 small interfering RNA (siRNA) and negative control siRNA (NC siRNA) were provided by HANBIO (Shanghai, China). The sequences of the siRNAs and the negative control used in the present study were as follows: FHL2 siRNA, 5′-GGA​CUU​GUC​CUA​CAA​GGA​UTT-3′ and 3′-AUC​CUU​GUA​GGA​CAA​GUC​CTT-1′; and negative control (NC siRNA), 5′-UUC​UCC​GAA​CGU​GUC​ACG​UTT-3′ and 3′-ACG​UGA​CAC​GUU​CGG​AGA​ATT-1′. H9C2 cardiac myocytes were cultured to 40–50% confluence and seeded at 1–3×10^5^ cells/well in 12 well plates. After incubating for 24 h at 37°C, the cells were transfected with 20 nm FHL siRNA or NC siRNA. Transfection was performed using Lipofectamine 2000 (Invitrogen) according to the manufacturer’s instructions. Western blotting was used to validate the transfection efficiency of FHL2 siRNA 48 h after transfection, and whole-cell extracts were collected to test the FHL2 and TGF-β1 contents.

### Statistical Analysis

All results are presented as the mean ± standard error of the mean (SEM). Statistical tests were conducted using the GraphPad Prism 9 software (San Diego, CA, United States). Differences between groups were compared using one-way ANOVA or unpaired Student’s *t*-test. The correlation between two variables was determined using Pearson’s correlation coefficient. The level of statistical significance was set at *p* < 0.05. All measurements were performed at least three times.

## Results

### QSYQ Alleviates Myocardium Injury and Reduces Cardiac Fibrosis

Heart failure presents as myocardial dysfunction, which was observed in the present study at 10 weeks after AAS surgery, as evidenced by the lower ejection fraction, fraction shortening, and reduced MBF in rats. Notably, treatment with QSYQ significantly restored heart function after AAS (Supplementary Figures S1A–E). Myocardial dysfunction reflects alterations in myocardial structure. As expected, AAS for 10 weeks resulted in myocardial hypertrophy, which led to an increase in the HW/BW; HW/TL; cardiomyocyte cross-sectional area; ANF/18S, BNP/18S, and Myh7/Myh6 mRNA levels; and plasma NT-ProBNP levels, which were also significantly suppressed by the QSYQ post-treatment (Supplementary Figures S2A–J) (*p < 0.05*). Interestingly, what was observed *in vivo* could be simulated *in vitro* because H9C2 cells exposed to Ang II responded similarly to rat cardiomyocytes after AAS in terms of changes in cell size and ANF/18S, BNP/18S, and Myh7/18S mRNA levels. QSYQ and its main ingredients inhibited Ang II-induced H9C2 cell hypertrophy (Supplementary Figures S3A–D) (*p < 0.05*).

**FIGURE 1:**
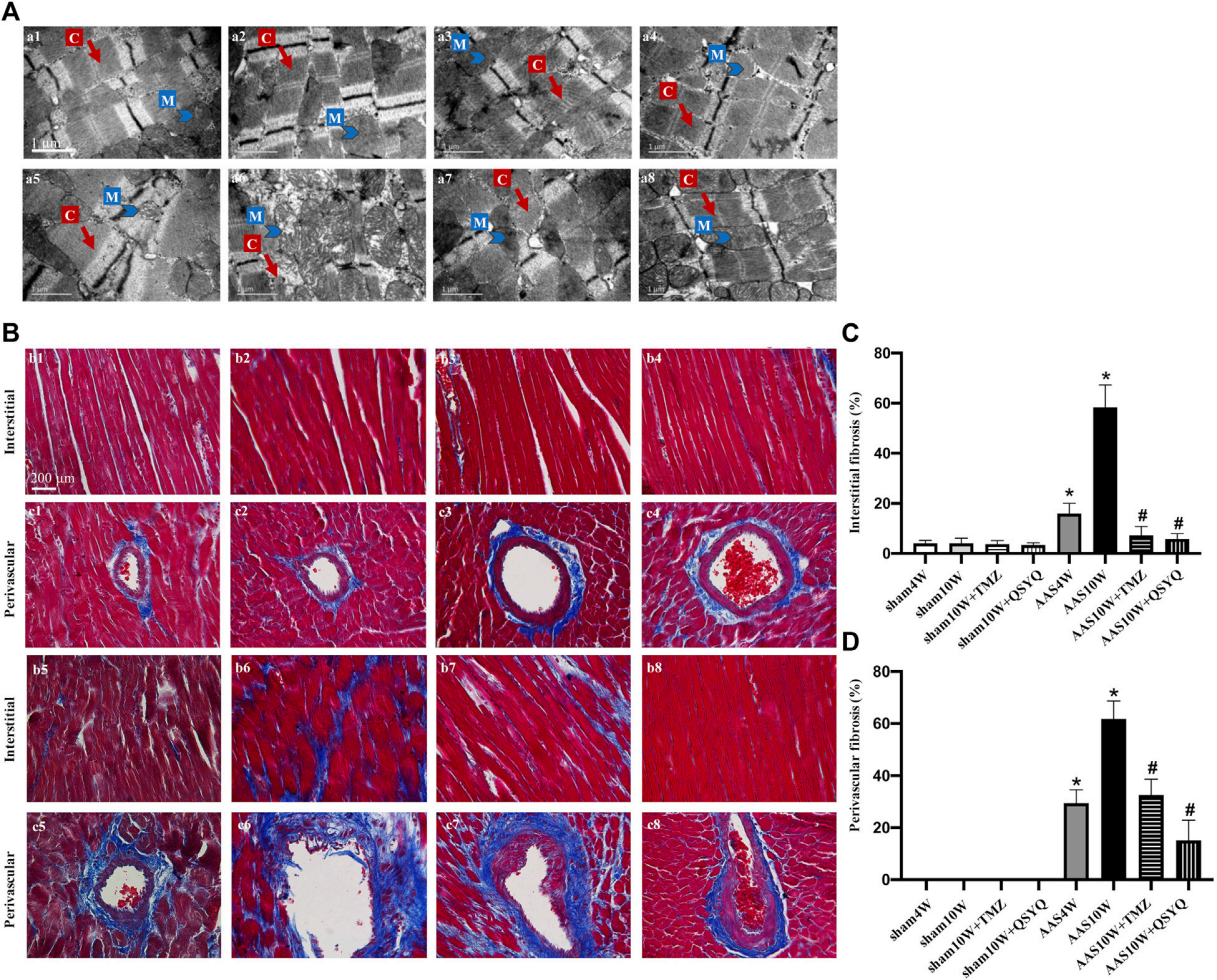
QSYQ post-treatment inhibited myocardial fiber rupture and cardiac fibrosis. **(A)** Effects of QSYQ post-treatment on the ultrastructure of myocardial tissues of rats in the Sham4W (a1), Sham10W (a2), Sham10W + TMZ (a3), Sham10W + QSYQ (a4), AAS4W (a5), AAS10W (a6), AAS10W + TMZ (a7), and AAS10W + TMZ (a8) groups. Scale bar = 1 μm. **(B)** Representative images of Masson’s trichrome staining in the Sham4W (b1 and c1), Sham10W (b2 and c2), Sham10W + TMZ (b3 and c3), Sham10W + QSYQ (b4 and c4), AAS4W (b5 and c5), AAS10W (b6 and c6), AAS10W + TMZ (b7 and c7), and AAS10W + TMZ (b8 and c8) groups. Scale bar = 200 μm. **(C,D)** Quantification results of interstitial fibrosis and perivascular fibrosis measured by Masson’s trichrome staining. Data are presented as the mean ± SEM, *n* = 3. ^*^ *p* < 0.05 vs. sham; ^#^ *p* < 0.05 vs. AAS. AAS, ascending aortic stenosis; C: cardiac myofibril; M: mitochondrial.

Next, we explored whether the QSYQ treatment inhibited cardiac injury and fibrosis after AAS. The transmission electron microscopy results showed that, compared with the sham groups, the AAS10W surgery resulted in prominent damage to cardiac myocytes, which manifested as ruptured myofibrils, swollen mitochondria, and edematous intermyofibril apace; however, this damage was improved by QSYQ treatment (Figure 1A). The protective role of QSYQ in myocardial mitochondria after AAS was confirmed by the finding that the level of ATP-5d, which encodes the delta subunit of mitochondrial ATP synthase, and levels of ATP/AMP and ATP/ADP were downregulated in the AAS10W group, while the QSYQ post-treatment significantly increased these energy-related parameters (Supplementary Figures S4A–D) (*p < 0.05*).

We then investigated whether the QSYQ treatment could reduce cardiac fibrosis. Masson’s trichrome staining (Figures 1B–D) and Sirius Red staining (Supplementary Figures S5A,B) were used to assess the degree of collagen deposition in both the interstitial and perivascular areas. The results showed that cardiac fibrosis was significantly enhanced in the AAS10W group compared with the sham group (*p < 0.05*). QSYQ post-treatment elicited a significant decrease in the average collagen volume after the AAS challenge (*p < 0.05*). Overall, QSYQ reduced cardiac fibrosis secondary to cardiac hypertrophy induced by pressure overload.

### QSYQ Ameliorates Myocardial Cell Apoptosis Both *In Vivo* and *In Vitro*

**FIGURE 2:**
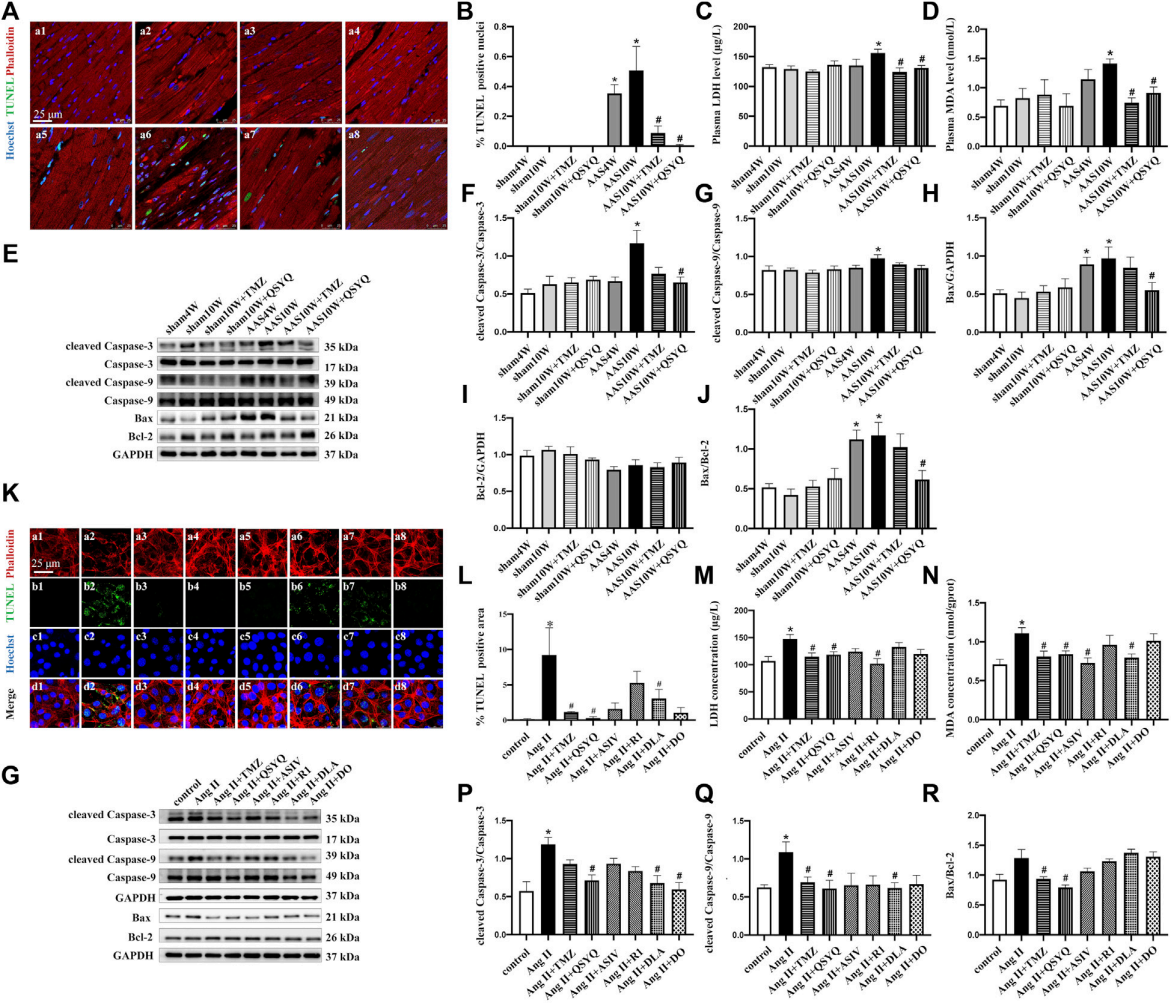
Effects of QSYQ and its main ingredients on cardiomyocyte apoptosis both *in vivo* and *in vitro*. **(A)** TUNEL staining of rat heart sections in the Sham4W (a1), Sham10W (a2), Sham10W + TMZ (a3), Sham10W + QSYQ (a4), AAS4W (a5), AAS10W (a6), AAS10W + TMZ (a7), and AAS10W + TMZ (a8) groups. Scale bar = 25 μm. **(B)** Quantification result of TUNEL-positive cardiomyocytes in rat hearts. Data are presented as the mean ± SEM, *n* = 3. **(C)** Serum lactate dehydrogenase (LDH) level detected by ELISA in different groups. **(D)** Serum malondialdehyde (MDA) level detected by ELISA in the different groups. **(E)** Western blot of apoptosis-related proteins: cleaved caspase-3/caspase-3, cleaved caspase-9/caspase-9, Bax, Bcl-2, and Bax/Bcl-2. **(F–J)** Quantification analyses of the Western blot results for cleaved caspase-3/caspase-3, cleaved caspase-9/caspase-9, Bax, Bcl-2, and Bax/Bcl-2. Data are presented as the mean ± SEM. *n* = 4. **(K)** TUNEL and phalloidin staining of H9C2 cells in the control (a1, b1, c1, and d1), Ang II (a2, b2, c2, and d2), Ang II + TMZ (a3, b3, c3, and d3), Ang II + QSYQ (a4, b4, c4, and d4), Ang II + ASIV (a5, b5, c5, and d5), Ang II + R1 (a6, b6, c6, and d6), Ang II + DLA (a7, b7, c7, and d7), and Ang II + DO (a8, b8, c8, and d8) groups. Bar = 25 μm. **(L)** Quantification result of TUNEL-positive cells. Data are presented as the mean ± SEM, n = 3. **(M)** Lactate dehydrogenase (LDH) level detected in supernatants of H9C2 cells by ELISA. **(N)** Malondialdehyde (MDA) level detected in H9C2 cell lysates by ELSA. Data are presented as the mean ± SEM, n = 4. **(O)** Western blot of apoptosis-related proteins in H9C2 cells from the different groups: cleaved caspase-3/caspase-3, cleaved caspase-9/caspase-9, Bax, and Bcl-2. **(P–R)** Quantification of the Western blot results for cleaved caspase 3/caspase 3, cleaved caspase-9/caspase 9, and Bax/Bcl-2 in H9C2 cells from the different groups. Data are presented as the mean ± SEM, *n* = 4. ^*^ *p* < 0.05 vs. control; ^#^ *p* < 0.05 vs. Ang II.

Sustained pressure overload ultimately triggers maladaptive cardiac hypertrophy and induces cardiomyocyte loss *via* apoptosis (Goh et al., 2019). In the present study, a TUNEL assay was performed to detect cell apoptosis, and the results are presented as the percentage of TUNEL-positive nuclei. As illustrated in Figures 2A,B, TUNEL-positive nuclei were significantly higher in the myocardium of AAS10W rats than in the sham rats, and the values were greatly diminished by the QSYQ post-treatment (*p < 0.05*). The effect of TMZ on apoptosis was similar to that of QSYQ. The serum LDH and MDA levels were also measured to assess the effects of QSYQ on myocardial injury, thus demonstrating that QSYQ significantly reduced both the serum LDH and heart MDA levels compared with that of AAS10W (Figures 2C,D) (*p* < 0.05). Collectively, the above results indicate that post-treatment with QSYQ ameliorated myocardial apoptotic injury induced by chronic pressure overload.

Apoptosis involves various proteolytic enzymes, including caspases (Moorjani et al., 2006). Hence, the protein levels of cleaved caspase-3 and cleaved caspase-9 were determined in rats from the different groups by Western blotting (Figures 2E–J). The results showed that the protein expression of cleaved caspase-3 and cleaved caspase-9 in the AAS10W group was significantly higher than that in the sham group (Figures 2F,G) (*p* < 0.05). The QSYQ treatment significantly decreased the cleaved caspase-3 content but not the cleaved caspase-9 content after AAS10W treatment (*p < 0.05*). TMZ had no effect on the elevated cleaved caspase-3 and caspase-9 levels. We also measured the levels of Bax and Bcl-2, which are two proteins related to apoptosis. As shown in Figures 2E,H–J, the protein levels of proapoptotic Bax and the ratio of Bax/Bcl-2 were significantly upregulated in the AAS10W group compared with the sham group, although these changes were significantly attenuated by the QSYQ post-treatment (*p* < 0.05). Collectively, the above results indicate that the post-treatment with QSYQ inhibited cardiac myocyte apoptosis induced by chronic pressure overload.

H9C2 cells exposed to Ang II were used to further verify the effect of QSYQ on cardiac myocyte apoptosis and identify its main ingredients. As shown in Figures 2K,L, Ang II induced myocardial filament destruction and cell apoptosis. Treatment with QSYQ and DO significantly suppressed apoptosis after applying Ang II, whereas ASIV, R1, and DLA did not have a significant effect (*p < 0.05*). Moreover, TMZ exhibited an effect similar to that of QSYQ. The LDH levels were significantly upregulated after Ang II stimulation and significantly decreased after treatment with QSYQ, TMZ, and R1 but not with ASIV, DLA, and DO (Figure 2M) (*p* < 0.05). The MDA levels were decreased compared with the Ang II group after treatment with QSYQ, TMZ, ASIV, and DLA but not with R1 and DO (Figure 2N) (*p* < 0.05). The protein levels of apoptosis-related proteins were assessed by Western blotting, which showed that the QSYQ, DLA, and DO treatments reduced the ratio of cleaved caspase-3 to caspase-3 induced by Ang II; however, the other ingredients did not have a significant effect (Figures 2O–R) (*p < 0.05*). Compared with the Ang II group, QSYQ and DLA inhibited the ratio of cleaved caspase-9 to caspase-9 (*p* < 0.05), and TMZ and QSYQ decreased the ratio of Bax to Bcl-2 (*p* < 0.05). The effects of QSYQ on the Ang II-induced impairment of ATP production in H9C2 cells were also observed (Supplementary Figures S6A,B). QSYQ significantly reversed Ang II-induced downregulation of ATP/ADP and ATP/AMP, whereas ASIV increased the ATP/ADP levels (*p* < 0.05). Mitochondrial respirometry of H9C2 cells was performed using 2-channel high-resolution Oxyraph-2k, and the results showed that QSYQ significantly improved the function of mitochondrial complex II and upregulated ATP production, which was impaired by Ang II (Supplementary Figures S6C,D) (*p* < 0.05). In summary, consistent with the *in vivo* experiment, QSYQ alleviated cardiomyocyte injury and cell apoptosis, and DLA was a major player.

### QSYQ Reduces the Release of Chemokine RP S19 From Apoptotic Cells and Inhibits Its Colocalization With the C5a Receptor

**FIGURE 3:**
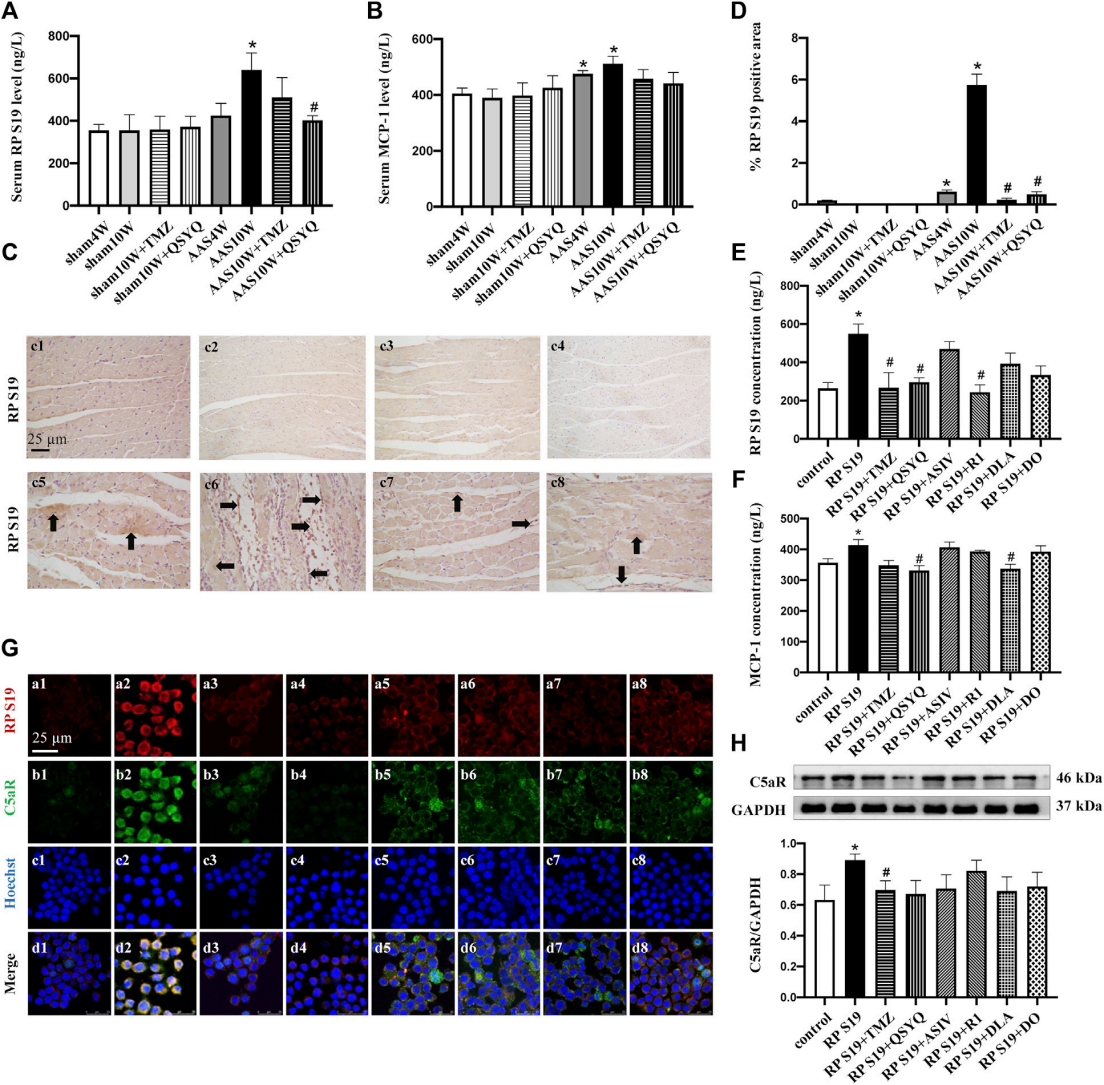
QSYQ reduced the release of chemokine RP S19 and inhibited its colocalization with the C5a receptor. **(A,B)** Rat serum RP S19 and MCP-1 levels detected by ELISA in rats from different groups. **(C)** Representative photomicrographs of immunohistochemistry staining for RP S19 in rat myocardium in the Sham4W (c1), Sham10W (c2), Sham10W + TMZ (c3), Sham10W + QSYQ (c4), AAS4W (c5), AAS10W (c6), AAS10W + TMZ (c7), and AAS10W + QSYQ (a8) groups. **(D)** Quantitative analysis results of RP S19 positive area. Arrows indicate the RP S19-positive area. Bar = 50 μm. Data are presented as the mean ± SEM, *n* = 3. **(E,F)** RP S19 and MCP-1 levels detected by ELISA in the H9C2 cell supernatants. Data are presented as the mean ± SEM, *n* = 4. **(G)** Immunofluorescence images of C5aR, RP S19 and Raw264.7 monocyte nucleus in the control (a1, b1, c1, and d1), RP S19 (a2, b2, c2, and d2), RP S19 + TMZ (a3, b3, c3, and d3), RP S19 + QSYQ (a4, b4, c4, and d4), RP S19 + ASIV (a5, b5, c5, and d5), RP S19 + R1 (a6, b6, c6, and d6), RP S19 + DLA (a7, b7, c7, and d7), and RP S19 + DO (a8, b8, c8, and d8) groups. Scale bar = 25 μm. Data are presented as the mean ± SEM, *n* = 3. **(H)** Western blotting analysis of Raw264.7 monocyte C5aR. Data are presented as the mean ± SEM, *n* = 4. ^*^ *p* < 0.05 vs. sham or control; ^#^ *p* < 0.05 vs. AAS or RP S19.

Apoptotic cells can release chemokine RP S19 to recruit monocytes from the circulation to the damaged myocardium (Yamamoto, 2000; Zheng et al., 2019). Thus, after pressure overload *in vivo* and Ang II stimulation *in vitro*, we assessed the effect of QSYQ and its main ingredients on RP S19 and MCP-1, a well-known chemokine critically involved in monocyte infiltration. As expected, the ELISA results revealed that the serum levels of RP S19 (Figure 3A) and MCP-1 (Figure 3B) were significantly upregulated in the AAS10W group (*p* < 0.05). Consistent with the ELISA results, immunohistochemical staining of RP S19 in AAS10W rat tissue also showed upregulated RP S19 expression (Figures 3C,D) (*p* < 0.05). QSYQ post-treatment had a pronounced inhibitory effect on RP S19 expression and inhibited the expression of MCP-1 both *in vivo* and *in vitro* (*p* < 0.05). The RP S19 (Figure 3E) and MCP-1 (Figure 3F) concentrations in Ang II-stimulated H9C2 cell supernatants significantly increased (*p* < 0.05). Interestingly, component R1 was found to inhibit the expression of RP S19 (Figure 3E), whereas DLA inhibited the expression of MCP-1 (Figure 3F), thus implying that the two components each exert an effect on a unique target.

We then examined the effect of QSYQ and its main ingredients on the C5a receptor (C5aR) using immunofluorescence and Western blotting. Raw264.7 monocytes were stimulated with RP S19 and treated with QSYQ and the main ingredients. Figures 3G,H show that QSYQ and all other ingredients except for DO inhibit the colocalization of RP S19 and C5aR but had no significant effect on the C5aR protein level. Collectively, these results suggest that the expression and release of the chemokine RP S19 and its colocalization with the C5a receptor can be reduced by the QSYQ treatment, which may contribute to its inhibitory effect on inflammatory cell infiltration.

### QSYQ Inhibits Monocyte Infiltration, Macrophage Polarization, and TGF-β1 Expression After AAS

**FIGURE 4:**
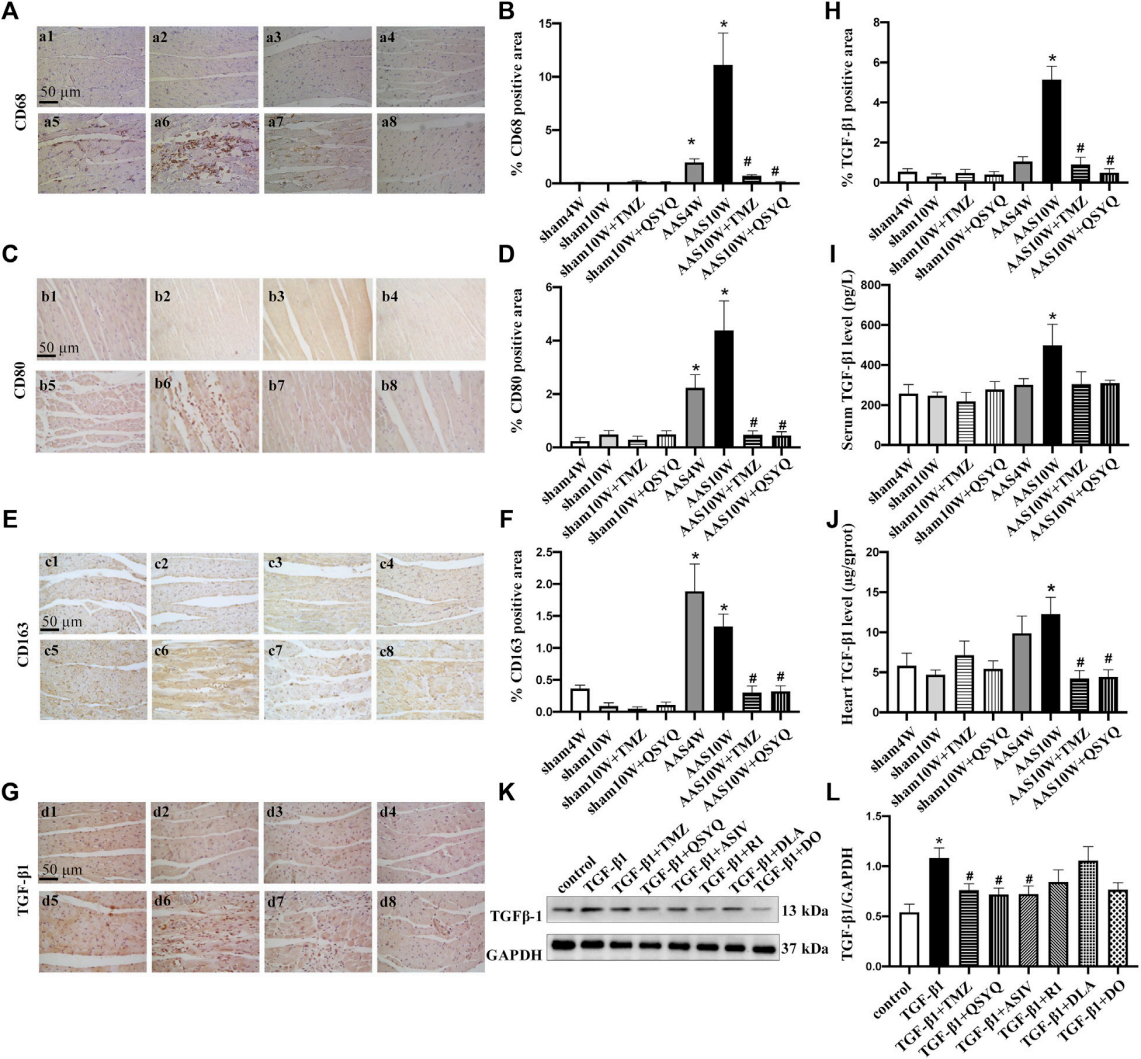
QSYQ inhibited monocyte infiltration, macrophage polarization and TGF-β1 expression. The four panels on the left show the immunohistochemistry staining results for CD68 **(A,B)**, CD80 **(C,D)**, CD163 **(E,F)**, and TGF-β1 **(G,H)** in the Sham4W (a1, b1, c1, and d1), Sham10W (a2, b2, c2, and d2), Sham10W + TMZ (a3, b3, c3, and d3), Sham10W + QSYQ (a4, b4, c4, and d4), AAS4W (a5, b5, c5, and d5), AAS10W (a6, b6, c6, and d6), AAS10W + TMZ (a7, b7, c7, and d7), and AAS10W + QSYQ (a8, b8, c8, and d8) groups in myocardium and its quantification analysis results, respectively. Bar = 50 μm. Data are presented as the mean ± SEM, *n* = 6. **(I)** Serum TGF-β1 level detected by ELISA. **(J)** TGF-β1 level in rat myocardial tissue detected by ELISA. Data are presented as the mean ± SEM, *n* = 4. **(K,L)** Protein level of TGF-β1 in Raw264.7 cells detected using Western blot. Data are presented as the mean ± SEM, *n* = 4. ^*^ *p* < 0.05 vs. sham or control; ^#^ *p* < 0.05 vs. AAS or RP S19.

Macrophages are extensively implicated in cardiac remodeling and fibrosis in HF and are associated with left ventricular pressure overload (Xia et al., 2009; Hulsmans et al., 2018). Therefore, we examined the potential effects of QSYQ treatment on monocyte infiltration and macrophage polarization. Immunohistochemical staining results of CD68 (monocyte marker) (Figures 4A,B), CD80 (M1-type macrophage marker) (Figures 4C,D), and CD163 (M2-type macrophage marker) (Figures 4E,F) showed that pressure overload induced monocyte infiltration and differentiation into macrophages, thus provoking macrophage M1/M2-type polarization (*p* < 0.05). Nevertheless, QSYQ significantly inhibited monocyte infiltration and macrophage polarization after the AAS10W surgery, and TMZ had a similar effect (*p* < 0.05).

TGF-β1 plays a critical role in the pathogenesis of cardiac remodeling and fibrosis (Jahanyar et al., 2007). Therefore, we measured the TGF-β1 expression levels both *in vivo* and *in vitro*. The *in vivo* results showed that TGF-β1 was dramatically upregulated in the rat hearts subjected to AAS challenge, as revealed by the immunohistochemical staining (Figures 4G,H) and ELISA results (Figures 4I,J) (*p* < 0.05). Nevertheless, the QSYQ treatment distinctly suppressed the augmentation of TGF-β1 in the heart (*p* < 0.05). Figures 4K,L showed an increase of TGF-β1 in Raw264.7 cells exposed to RP S19, which was inhibited by ASIV and QSYQ (*p* < 0.05).

### QSYQ and Its Main Ingredients Inhibits TGF-β1-Induced TGFβR II and Smads Protein Expression in RDF Myofibroblasts

**FIGURE 5:**
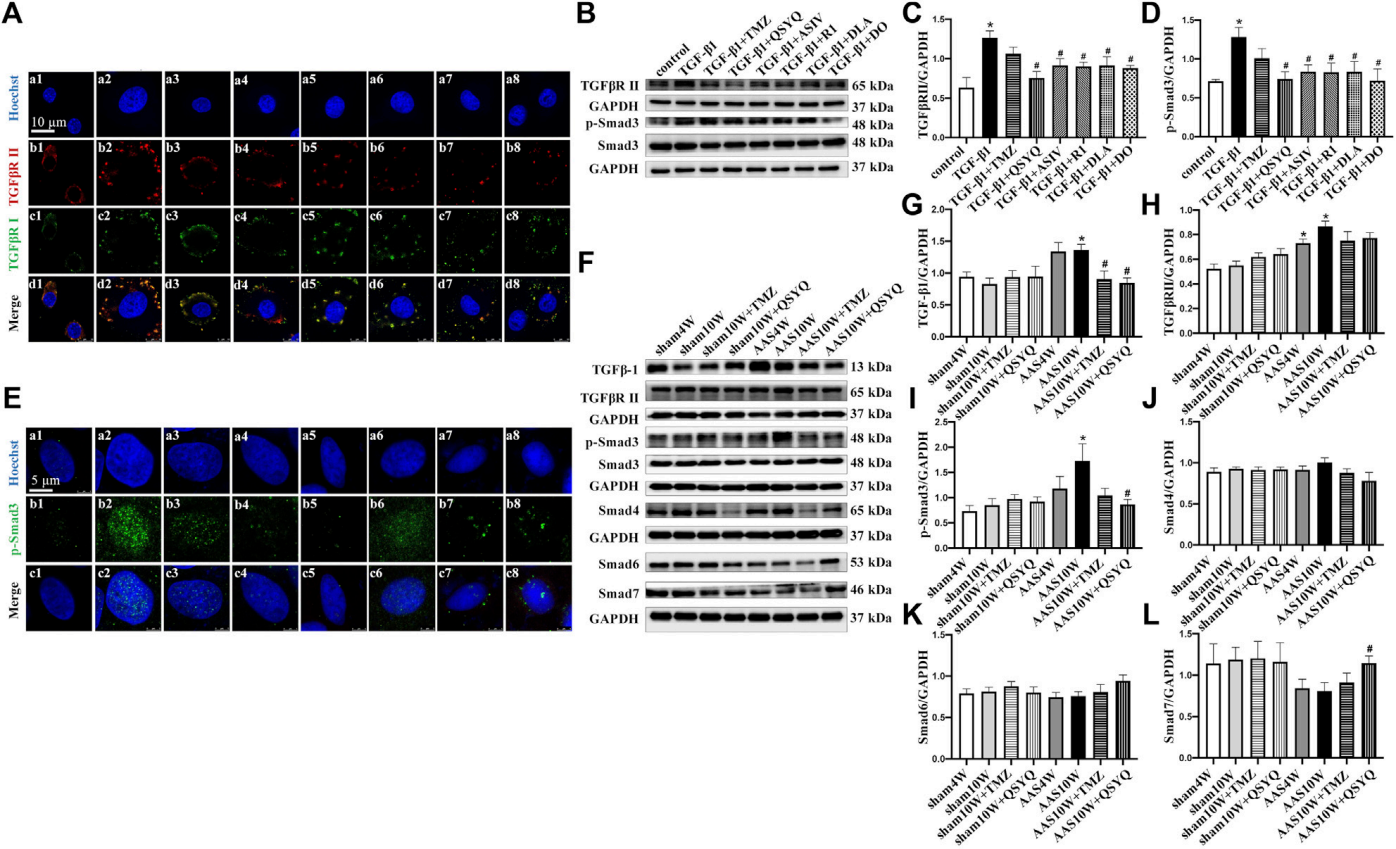
QSYQ inhibited AAS-induced increases of TGF-β1 and its receptor TGFβR II as well as activation of Smads. **(A)** Immunofluorescence images of TGFβR II, TGFβR I, and nucleus in RDF cells in the control (a1, b1, c1, and d1), TGF-β1 (a2, b2, c2, and d2), TGF-β1+TMZ (a3, b3, c3, and d3), TGF-β1+QSYQ (a4, b4, c4, and d4), TGF-β1+ASIV (a5, b5, c5, and d5), TGF-β1+R1 (a6, b6, c6, and d6), TGF-β1+DLA (a7, b7, c7, and d7), and TGF-β1+DO (a8, b8, c8, and d8) groups. **(B)** Expression of TGFβR II, p-Smad3, and Smad3 in RDF cells examined by Western blot. **(C,D)** Quantification of the Western blot results for TGFβR II and p-Smad3/Smad3. Data are presented as the mean ± SEM, *n* = 4. **(E)** Immunofluorescence images of p-Smad3 and nuclei in RDF cells. Scale bar = 25 μm. **(F)** Expression of TGF-β1, TGFβR II, p-Smad3, Smad4, Smad6, and Smad7 in rat myocardium examined by Western blot. **(G–L)** Quantification of the Western blot results for TGF-β1, TGFβR II, p-Smad3/Smad3, Smad4, Smad6, and Smad7 in Panel 5E. Data are presented as the mean ± SEM, *n* = 4. ^*^ *p* < 0.05 vs. sham or control; ^#^ *p* < 0.05 vs. AAS or TGF-β1.

TGF-β1 binds to its type I (TGFβR I) and type II (TGFβR II) receptors, thereby triggering phosphorylation and nuclear translocation of Smad2 and Smad3 and inducing profibrotic gene transcription (Shi and Massague, 2003; Khalil et al., 2017). Thus, we detected the protein levels of TGFβR II and p-Smad3 (Figures 5A–D) as well as the nuclear translocation of p-Smad3 in RDF myofibroblasts (Figure 5E). The cells were treated with QSYQ and its main ingredients and then stimulated with TGF-β1. TGF-β1 increased the expression of TGFβR II and p-Smad3 (Figures 5B–D), which was significantly decreased by QSYQ and the four main ingredients (Figures 5B–D) (*p* < 0.05). In addition, QSYQ inhibited the nuclear translocation of p-Smad3, and the main components exhibited similar effects, with R1 being the least effective (Figure 5E).

Consistent with the above results, the *in vivo* study showed that the protein levels of TGFβR II and p-Smad3 were significantly upregulated after the AAS10W treatment (Figures 5F–I) (*p* < 0.05). The QSYQ treatment significantly inhibited p-Smad3 expression and tended to lower the expression of TGFβR II.

Smad6 and Smad7 are well-known inhibitory Smads that inhibit TGF-β signaling by interfering with Smad4 activation. Intriguingly, we found that QSYQ markedly upregulated the protein levels of Smad7 after the AAS challenge compared with AAS10W (Figure 5L) (*p* < 0.05). In summary, QSYQ inhibited the expression of TGFβR II and p-Smad3 and upregulated the protein levels of Smad7.

### QSYQ Improves Collagen Degradation System Disorder Both *In Vivo* and *In Vitro*

**FIGURE 6:**
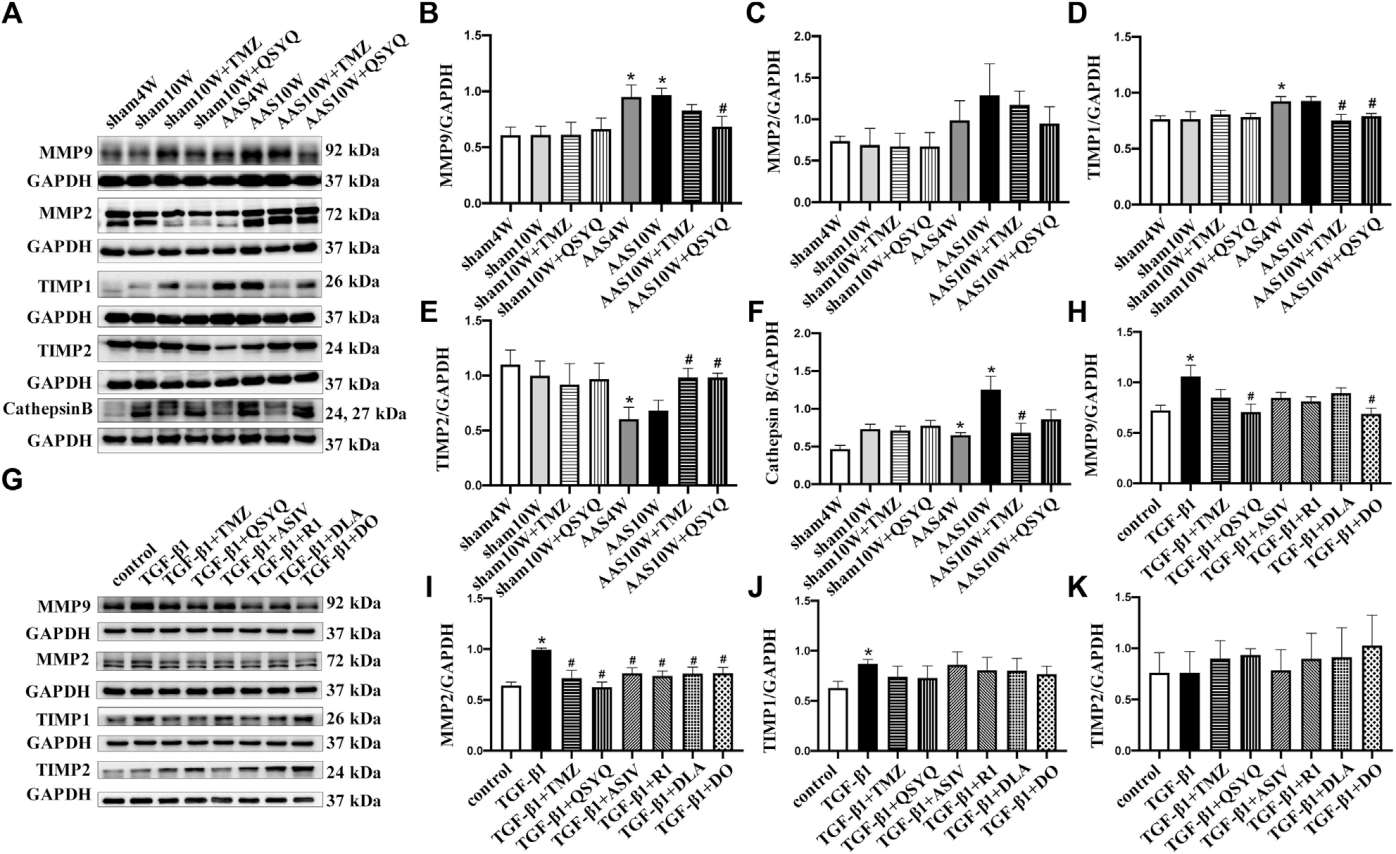
QSYQ regulated abnormal expression of MMP/TIMP. **(A)** Expression of MMP2, MMP9, TIMP1, TIMP2, and cathepsin B in rat myocardium examined by Western blotting. **(B–F)** Quantification of Western blot results of MMP2, MMP9, TIMP1, TIMP2, and cathepsin **(B)**. **(G)** Expression of MMP2, MMP9, TIMP1, and TIMP2 in RDF cells examined by Western blot. **(H–K)** Quantification of Western blot results for MMP2, MMP9, TIMP1, and TIMP2. Data are presented as the mean ± SEM, n = 4. ^*^ *p* < 0.05 vs. sham or control; ^#^ *p* < 0.05 vs. AAS or TGF-β1.

In addition to TGF-β1 signaling, which mediates collagen synthesis, matrix metalloproteinases (MMP2 and MMP9), tissue inhibitor of metalloproteinases (TIMP) (Li and Feldman, 2001), and cathepsin B participate in the regulation of cardiac fibrosis by controlling collagen degradation (Morrone et al., 2021). Therefore, we measured the protein levels of MMP2, MMP9, TIMP1, TIMP2, and cathepsin B *in vivo*. As shown in Figures 6A–F, the AAS challenge upregulated the expression of MMP9 (Figure 6B) and TIMP1 (Figure 6D) but significantly downregulated the expression of TIMP2 (Figure 6E), which was inhibited by QSYQ post-treatment (*p* < 0.05). The AAS challenge also upregulated the expression of cathepsin B (Figure 6F), which was inhibited by the TMZ treatment (*p* < 0.05). In line with the *in vivo* results, QSYQ markedly suppressed the expression of MMP9 (Figure 6H) and MMP2 (Figure 6I) in RDF cells stimulated by TGF-β1 (*p* < 0.05). All the effective ingredients downregulated the expression of MMP2, while only DO showed an inhibitory effect on MMP9 after TGF-β1 stimulation (Figures 6G–I) (*p* < 0.05). These results suggest that post-treatment with QSYQ restored the balance of the collagen degradation system impaired by pressure overload stress.

### QSYQ Inhibits Collagen Production Both *In Vivo* and *In Vitro*

**FIGURE 7:**
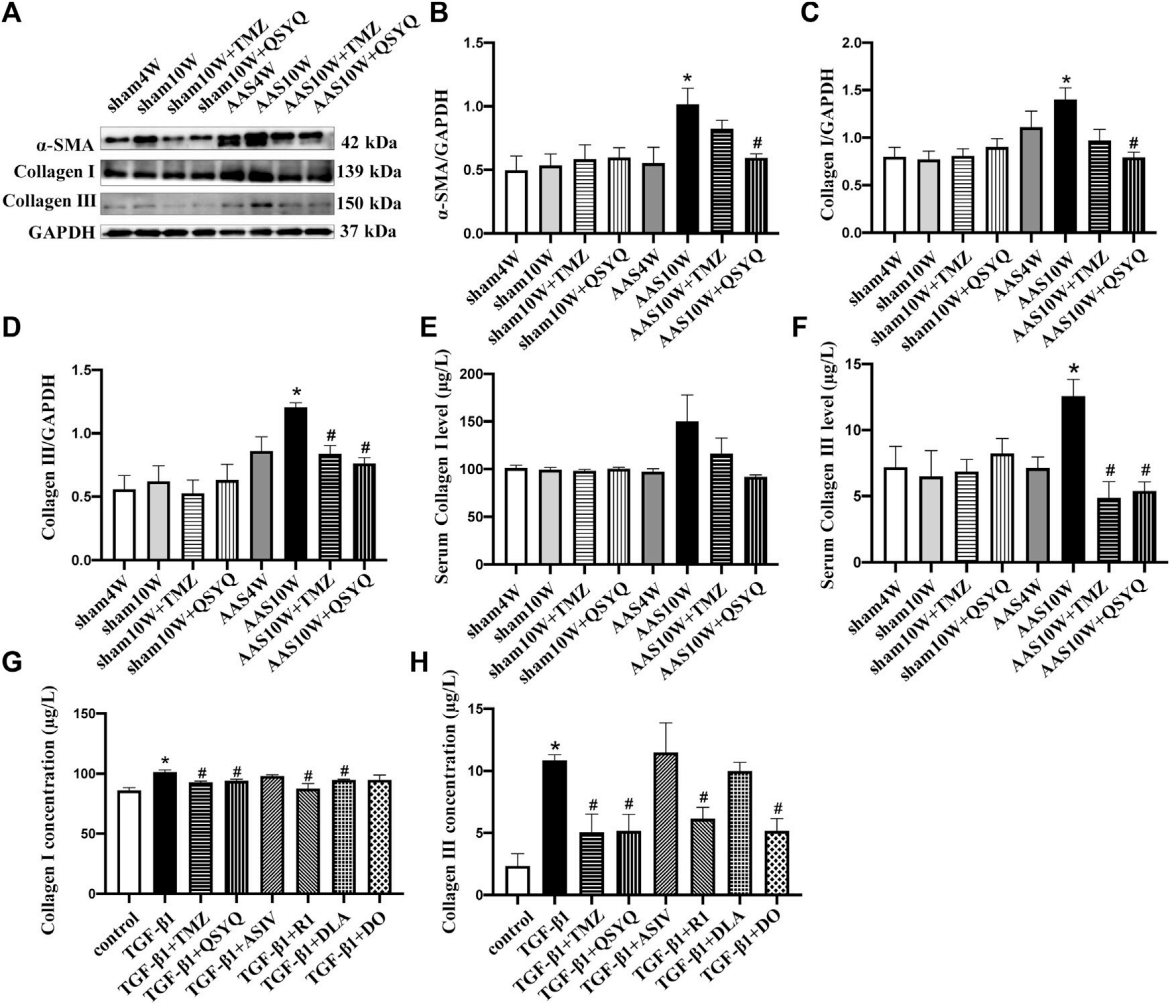
QSYQ ameliorated collagen production both *in vivo* and *in vitro*. **(A)** Expression of α-SMA, collagen I and collagen III in rat myocardium examined by Western blot. **(B–D)** Quantification of the Western blot results for α-SMA, collagen I and collagen III. Data are presented as the mean ± SEM, *n* = 4. **(E,F)** Serum collagen I and collagen III levels detected by ELISA. **(G,H)** Collagen I and collagen III levels in TGF-β1-stimulated RDF cell supernatants examined by ELISA. Data are presented as the mean ± SEM, *n* = 6. ^*^ *p* < 0.05 vs. sham or control; ^#^ *p* < 0.05 vs. AAS or TGF-β1.

These results suggest that QSYQ post-treatment may repress collagen production and deposition, even after cardiac hypertrophy is induced by pressure overload stress. To test this hypothesis, we examined the collagen expression in rats under various conditions. Western blotting showed that the QSYQ post-treatment significantly restored the *α*-SMA, collagen I, and collagen III protein levels in heart tissue compared to that of AAS10W (Figures 7A–D) (*p* < 0.05). The ELISA results showed that QSYQ significantly decreased the serum collagen III levels (Figures 7E,F) (*p* < 0.05). The QSYQ, DLA, and R1 treatment markedly decreased the collagen I concentration *in vitro* in the supernatants of TGF-β1 stimulated RDF cells, whereas QSYQ, R1, and DO significantly decreased the collagen III concentration (Figures 7G,H) (*p* < 0.05). These results suggested that QSYQ and its main ingredients inhibited collagen production.

### QSYQ Inhibits TGF-β1 Production in Cardiac Myocytes in a FHL2-Dependent Manner

**FIGURE 8:**
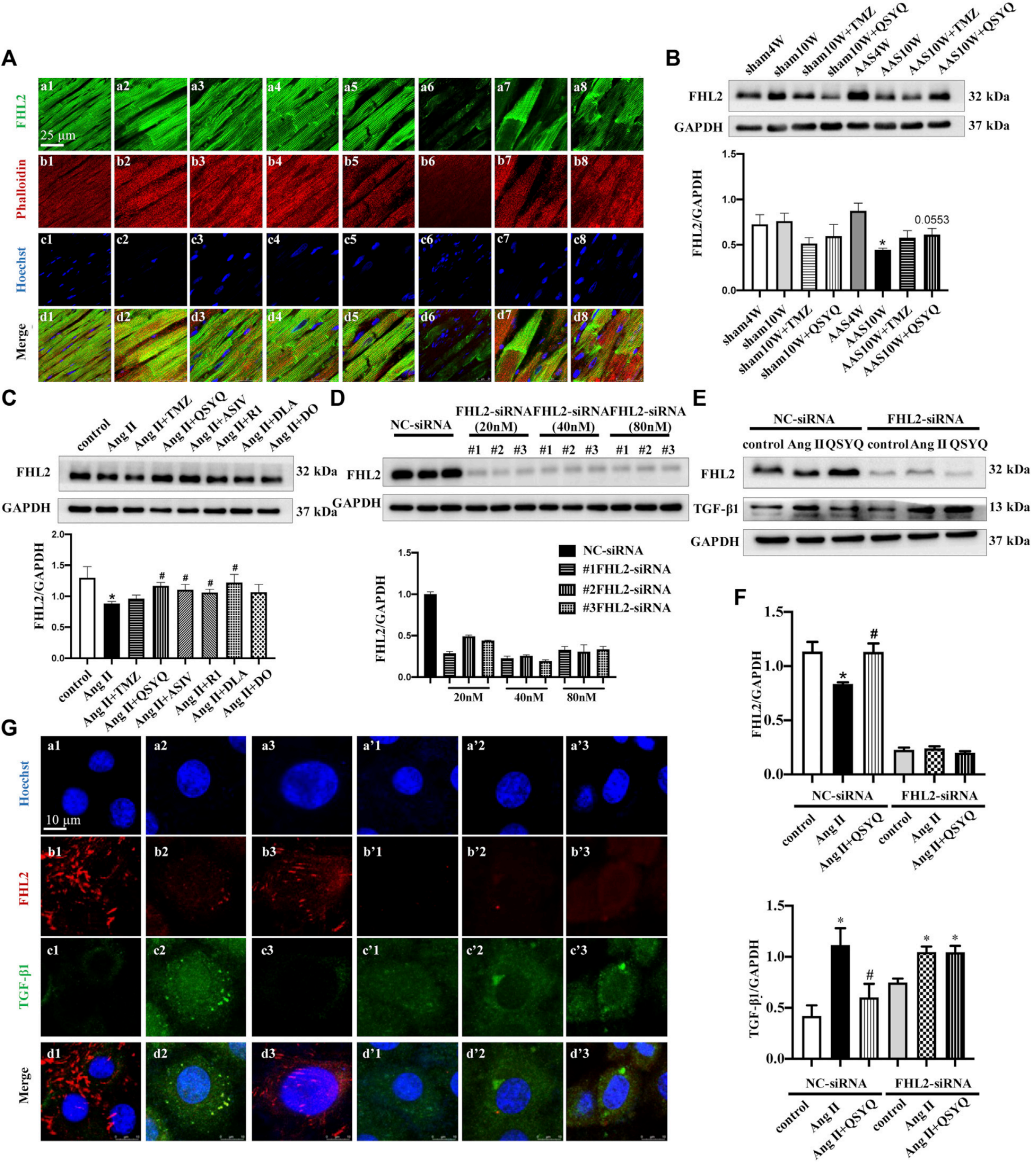
QSYQ inhibited cardiac myocyte release of TGF-β1 in a FHL2 dependent manner. **(A)** Immunofluorescence images of FHL2, phalloidin, and nuclei in rat myocardium in the Sham4W (a1, b1, c1, and d1), Sham10W (a2, b2, c2, and d2), Sham10W + TMZ (a3, b3, c3, and d3), Sham10W + QSYQ (a4, b4, c4, and d4), AAS4W (a5, b5, c5, and d5), AAS10W (a6, b6, c6, and d6), AAS10W + TMZ (a7, b7, c7, and d7), and AAS10W + QSYQ (a8, b8, c8, and d8) groups. Bar = 25 μm. **(B)** Expression of FHL2 in rat myocardium examined by Western blot with quantification shown below. **(C)** Expression of FHL2 in 1 µM Ang II stimulated H9C2 cells examined by Western blot, with quantification shown below. **(D)** Expression of FHL2 in FHL2 knock-downed H9C2 cells using siRNA examined by Western blot, with quantification shown below. **(E)** Expression of FHL2 and TGF-β1 in FHL2 knock-down H9C2 cells examined by Western blot. **(F)** Quantification of the results presented previously. Data are presented as the mean ± SEM, *n* = 4. ^*^ *p* < 0.05 vs. sham or control; ^#^ *p* < 0.05 vs. AAS or Ang II. **(G)** Immunofluorescence images of FHL2, TGF-β1, and nucleus in H9C2 cells in NC-siRNA: NC (a1, b1, c1, and d1), Ang II (a2, b2, c2, and d2), Ang II + QSYQ (a3, b3, c3, and d3) and FHL2-siRNA: NC (a’1, b’1, c’1, and d’1), Ang II (a’2, b’2, c’2, and d’2), and Ang II + QSYQ (a’3, b’3, c’3, and d’3). Scale bar = 25 μm.

In a study on the production of TGF-β1 after chronic pressure overload stress, large numbers of phalloidin-labeled cardiac myocytes were found to colocalize with TGF-β1 (Supplementary Figure S7A). In addition, H9C2 cells released TGF-β1 in response to Ang II stimulation, which was inhibited by QSYQ (Supplementary Figure S7B) (*p* < 0.05). Therefore, we explored the mechanism by which cardiac myocytes express TGF-β1. Our previous proteomic results (Chen et al., 2015) suggested that FHL2 may mediate the anti-TGF-β1 effects of QSYQ on cardiac myocytes. To verify this supposition, we first determined the expression level of FHL2 in our model and the possible effects of QSYQ. Both immunofluorescence (Figure 8A) and Western blotting (Figure 8B) results showed that FHL2 was upregulated at 4 weeks after AAS but reduced significantly after chronic pressure overload stress for 10 weeks. QSYQ post-treatment showed a trend of restoring FHL2 expression, although the difference was not statistically significant. In line with these results, the *in vitro* study showed that QSYQ and its main ingredients except for DO upregulated FHL2 in Ang II-stimulated H9C2 cells (Figure 8C) (*p* < 0.05). To further clarify the role of FHL2 in the effects of QSYQ, FHL2 was knocked down using siRNA. Figure 8D shows that FHL2 expression was significantly inhibited by siRNA, indicating successful knockdown (*p* < 0.05). Next, H9C2 cells were stimulated with Ang II. Both the Western blotting (Figures 8E,F) and immunofluorescence (Figure 8G) results showed that Ang II stimulation resulted in the upregulation of TGF-β1 and reduction of FHL2, which was reversed by the QSYQ treatment (*p* < 0.05). However, after FHL2 knockdown, QSYQ failed to inhibit TGF-β1 production. These results demonstrated that FHL2 mediated the protective effect of QSYQ on TGF-β1 in cardiac myocytes.

## Discussion

In the present study, we demonstrated that treatment with QSYQ ameliorates cardiac fibrosis after a pressure overload challenge. This effect was likely mediated by inhibition of myocardial apoptosis, cardiomyocyte RP S19 release, monocyte recruitment, TGF-β1 release, and the fibroblast TGF-β1/Smad signaling pathway. In addition, we found for the first time that QSYQ inhibited cardiomyocyte TGF-β1 production by restoring FHL2 expression.

Compensative left ventricle hypertrophy initially develops in response to pressure overload, which is followed by a state in which the left ventricle contractile performance is impaired, ultimately leading to heart failure. Myocardial fibrosis is the key determinant of the transition between the two episodes. Our previous study demonstrated that QSYQ is a potent anti-hypertrophic agent, and its effect is attributable to its capacity to counteract oxidative stress and impaired energy metabolism (Chen et al., 2015). The present study showed that 0.8 g/kg QSYQ (5 times equivalent to clinical dose), as a high dose, can protect against myocardial fibrosis that emerges after pressure overload-induced cardiac hypertrophy. However, it is critical to have a dose–response curve in the experimental setup that includes the usual dose in humans (Heinrich et al., 2020), while the dosage of QSYQ in the present study is selected based on our previous study (Chen et al., 2015).

The pathogenesis of pressure overload-induced myocardial fibrosis is a complex process involving a large number of cell types, cytokines, and signaling pathways. However, the mechanism underlying this process has not been clarified. Moreover, TGF-β1 has been well recognized to play a central role in the development of pressure overload provoked myocardial fibrosis. TGF-β1 is present as a latent complex in normal heart cells, such as macrophages and endothelial cells, and activated by a number of mediators, including RP S19, a component of the ribosome that is released by apoptotic cells and acts as a monocyte-selective chemoattractant (Nishiura et al., 1996; Horino et al., 1998). Once activated, TGF-β1 binds to TGFβR II on the cell surface, phosphorylates the cytoplasmic domain of TGFβR I, and exerts profibrotic actions *via* Smads. Activation of the Smad3 signaling pathway stimulates the transformation of fibroblasts to myofibroblasts and enhances the synthesis of extracellular matrix protein. Our previous study showed the involvement of RP S19-mediated TGF-β1 signaling in I/R-induced myocardial fibrosis (Zheng et al., 2019). In the current study, we found that RP S19 was upregulated and released after 10 weeks of AAS challenge, and this change was accompanied by increased TGF-β1 expression. Consistent with the *in vivo* results, our *in vitro* experiments showed that Ang II stimulated RP S19 release from cardiomyocytes, which in turn enhanced TGF-β1 expression in RAW264.7 cells. These results suggest that the reaction cascade is similar to that observed during the progression of myocardial fibrosis, regardless of the trigger. More importantly, QSYQ attenuated all insults caused by pressure overload *in vivo* and Ang II *in vitro*, suggesting that QSYQ is an optimal option for myocardial fibrosis induced by different stresses.

In addition, we found that FHL2 was significantly downregulated after AAS and QSYQ was remarkably upregulated. Knockdown of FHL2 using siRNA led to the failure of QSYQ to inhibit the upregulation of TGF-β1 induced by Ang II, thus highlighting the crucial protective role of FHL2 on the upregulation of TGF-β1. FHL2 belongs to the LIM-domain only proteins, and the absence of FHL2 exaggerated isoproterenol-induced cardiac hypertrophy (Kong et al., 2001). A previous study revealed that deficiency of FHL2 aggravates liver fibrosis in mice (Huss et al., 2013), where it negatively regulates TGF-β1 expression (Dahan et al., 2017). Nonetheless, the mechanism by which FHL2 regulates fibrosis has not been clarified. Purcell et al. reported that FHL2 binds to and inhibits extracellular signal-regulated kinase (ERK) in cardiomyocytes, thereby preventing ERK-induced cardiac hypertrophy (Purcell et al., 2004). Whereas a study by Duan et al. showed that FHL2 regulates β-catenin, which plays an important role in TGF-β1-induced fibroblast activation (Duan et al., 2020). How QSYQ interferes with FHL2 signaling and thus affects the expression of TGF-β1 is currently unknown and requires further study.

Myocardial fibrosis following pressure overload is a consequence of multiple insults, including energy metabolism disorders and the resultant ATP depletion, oxidative stress, cell injury, and apoptosis. Effective management of myocardial fibrosis should be applied to multiple targets. In the present study, we evaluated the effect of the four major ingredients of QSYQ and found that DLA contributes to the protective effect of QSYQ on apoptosis, ASIV is responsible for retention of energy metabolism, and R1 protects cells from injury (Table 1). These results demonstrate the potential of QSYQ as a compound in Chinese medicine.

**TABLE 1:** Targets of QSYQ and its main ingredients on pressure overload-induced cardiac fibrosis.

Drug		QSYQ	ASIV	R1	DLA	DO
Target						
Energy	ATP/ADP	*	*	—	—	—
	ATP/AMP	*	—	—	—	—
Apoptosis	Cleaved caspase-3	*	—	—	*	*
	Cleaved caspase-9		—	—	*	—
	LDH	*	—	*	—	—
	MDA	*	*		*	—
Chemokines	RP S19	*	—	*		—
	MCP-1	*	—	—	*	—
Fibrosis	TGF-β1	*	*	—	—	—
	TGFβR II	*	*	*	*	*
	p-Smad3	*	*	*	*	*
	Smad7	*	—	—	—	—
	MMP2	*	*	*	*	*
	MMP9	*	—	—	—	*
	TIMP1	*	—	—	—	—
	TIMP2	*	—	—	—	—
	Collagen I	*	—	*	*	—
	Collagen III	*	—	*		*
	FHL2	*	*	*	*	—

*p < 0.05.

## Conclusion

The present study demonstrated that the high dose of QSYQ (0.8 g/kg) inhibited cardiac fibrosis after pressure overload-induced cardiac hypertrophy. This effect was related to the inhibition of myocardial apoptosis, cardiomyocyte RP S19 release, monocyte recruitment, TGF-β1 release, and the fibroblast TGF-β1/Smad signaling pathway. In addition, this study showed for the first time that QSYQ inhibited cardiomyocyte TGF-β1 production through the restoration of FHL2 expression. The results of the present study provided novel insights for a better understanding of the mechanism underlying the beneficial role of QSYQ in protecting myocardial fibrosis. However, dose-dependent effect of QSYQ on inhibition of cardiac fibrosis needs to be examined, and clinical studies are needed to verify whether QSYQ inhibits cardiac fibrosis after cardiac hypertrophy.

## Data Availability

The original contributions presented in the study are included in the article/Supplementary Materials; further inquiries can be directed to the corresponding authors.
