# Supplementary material for: QiShenYiQi Pill Ameliorates Cardiac Fibrosis After Pressure Overload-Induced Cardiac Hypertrophy by Regulating FHL2 and the Macrophage RP S19/TGF-β1 Signaling Pathway
Source: Front Pharmacol. 2022 Jul 13;13:918335. doi: 10.3389/fphar.2022.918335 (PMC9326396; doi:10.3389/fphar.2022.918335)
Supplement: Supplementary file 3 [file DataSheet1.pdf]

Supplementary Material

1. Supplementary Methods

1.1 Echocardiographic analysis

Left ventricular function was evaluated 4 or 10 weeks after AAS using transthoracic echocardiography and a high-resolution echocardiography imaging system (VisualSonics, VEVO2100 & VINNO, X10-23L). Briefly, rats were anesthetized with 1.5–2.0% isoflurane by mask, the chest was shaved, the animals were placed in a supine position on a warming pad, and electrocardiogram limb electrodes were attached. Two-dimensional cine loops and guided M-mode frames were recorded from the parasternal short- and long-axes. Echocardiography was performed by a single experienced operator in a blind manner. All data were analyzed offline at the end of the study using software included with the ultrasound system. All measurements represent the mean of three consecutive cardiac cycles.

1.2 Myocardial blood flow

Following the echocardiographic analysis, the rats underwent thoracotomy under anesthesia, and the myocardial blood flow (MBF) was measured using a laser Doppler perfusion imager (PeriScan PIM3, Perimed, Sweden) equipped with a computer. All images were evaluated using the software LDPIwin 3.1 (Perimed, Sweden). For each rat, the MBF was the average of three independent measurements.

1.3 Body weight/heart weight and body weight/tibia length

The animals were sacrificed after the MBF evaluation, and their hearts were removed and washed with normal saline. Body weight (BW) and heart weight (HW) were determined, and tibia length (TL) was measured using a Vernier caliper. The ratios of HW to BW (HW/BW) and HW to TL (HW/TL) were then calculated to evaluate the cardiac hypertrophic response to the procedure.

1.4 Real-time quantitative PCR

Real-time quantitative PCR was performed to detect the mRNA levels of ANF, BNP, Myh6, Myh7, RP S19, MCP-1, and 18S rRNA from each sample. Total RNA was isolated from rat hearts or RDF myofibroblasts using TransZol Up reagent (TransGen Biotech, Beijing, China) according to the manufacturer's protocol. Then, the mRNA was reverse-transcribed into cDNA using a Hifair™ II 1st Strand cDNA Synthesis Super Mix for qPCR kit (Yeasen Biotechnology, Shanghai, China). Quantitative PCR analysis was performed using the Hieff UNICON® Power qPCR SYBR Green Master Mix (Yeasen Biotechnology, Shanghai, China). The primer sequences (all Rattus) were as follows: F-ANF: CTGCTTCGGGGGTAGGATTG, R-ANF: TTCGGTACCGGAAGCTGTTG; F-BNP: TCAAAGGACCAAGGCCCTAC, R-BNP: CGATCCGGTCTATCTTCTGCC; F-Myh6: AAGAGTGACAGGATGACGGA, R-Myh6: TAGGCGCTCCTTCTCTGACT; F-Myh7: CAACCTGTCCAAGTTCCGCA, R-Myh7: CCTAAGGTGCTGTTTCAAAGGC; F-RP S19: GACGTTAACCAGCAGGAGTT, R-RP S19: TCCTCCGTAGATCTTGGTCAT; F-MCP-1: TTCACAGTTGCTGCCTGTAG, R-MCP-1: ACTTCTGGACCCATTCCTTATTG; F-18S: CCCATTCGAACGTCTGCCCTATC and R-18S: TGCTGCCTTCCTTGGATGTGGTA. The following parameters were used for the detection of these sequences: 95 °C for 2 min, followed by 40 cycles of 95 °C for 3 s for denaturation and 60 °C for 30 s for annealing and extension. The results were analyzed using an Agilent AriaMx Real-Time PCR System (Agilent Technologies, Penang, Malaysia). Gene expression levels were standardized based on the expression levels of the 18S gene.

1.5 NT-ProBNP measurement

The plasma NT-ProBNP levels in rat serum were measured using an ELISA kit (Andygene, Beijing, China) according to the manufacturer’s instructions.

1.6 Mitochondrial oxygen consumption assay

Mitochondrial oxygen consumption was measured polarographically at 37 °C in Mir05 buffer (0.5 mM EGTA-free acid, 3 mM $MgCl_2$, 110 mM D-sucrose, 60 mM lactobionic acid, 20 mM taurine, 20 mM HEPES free acid, 10 mM KH_2PO_4, and 1 g/L fatty acid free bovine serum albumin (BSA), pH 7.1) in a 2-channel high-resolution Oxyraph-2k (Oroboros Instruments, Innsbruck, Austria). Sample, substrates, inhibitors, and uncouplers were injected in the following order: ① living cells; ② 3.75 μg/mL digitonin; ③ 2 mM malate; ④ 2.5 mM ADP; ⑤ 10 μM cytochrome C; ⑥ 10 mM glutamate; ⑦ 10 mM succinate; ⑧ carbonycyanide 4-(trifluoromethoxy) phenylhydrazone (FCCP) titration to maximum respiration; ⑨ 0.513 μM rotenone; and ⑩ 2.5 μM antimycin A. Baseline values for oxygen (O_2) consumption were measured following the addition of each substrate or inhibitor. The basal O_2 consumption rate (state routine: living cell respiration), maximal O_2 consumption rate (state OXYPHOS: ADP stimulated respiration), uncoupled O_2 consumption rate (state ET: non-coupled respiration), and nonmitochondrial O_2 consumption rate (state ROX: residual oxygen consumption) were measured. Data acquisition and analysis were performed using Oxygraph-2k-Datlab 4.3.1.15 software (Oroboros Instruments, Innsbruck, Austria).

1.7 Cell counting kit-8 assay

Cell viability was assessed by a cell counting kit-8 (CCK-8, Dojindo Molecular Technologies, Gaithersburg, MD). Cells were seeded in a 96-well plate with 10000 cells/well. After stimulation, the CCK-8 solution was added to the culture medium, and the cultures were incubated for 1 h at 37 °C in humidified 95% air and 5% CO2. The absorbance was measured at 450 nm using a Microplate Reader (Bio-Tek, Vermont, America).

2. Supplementary Figures

Supplementary Figure 1

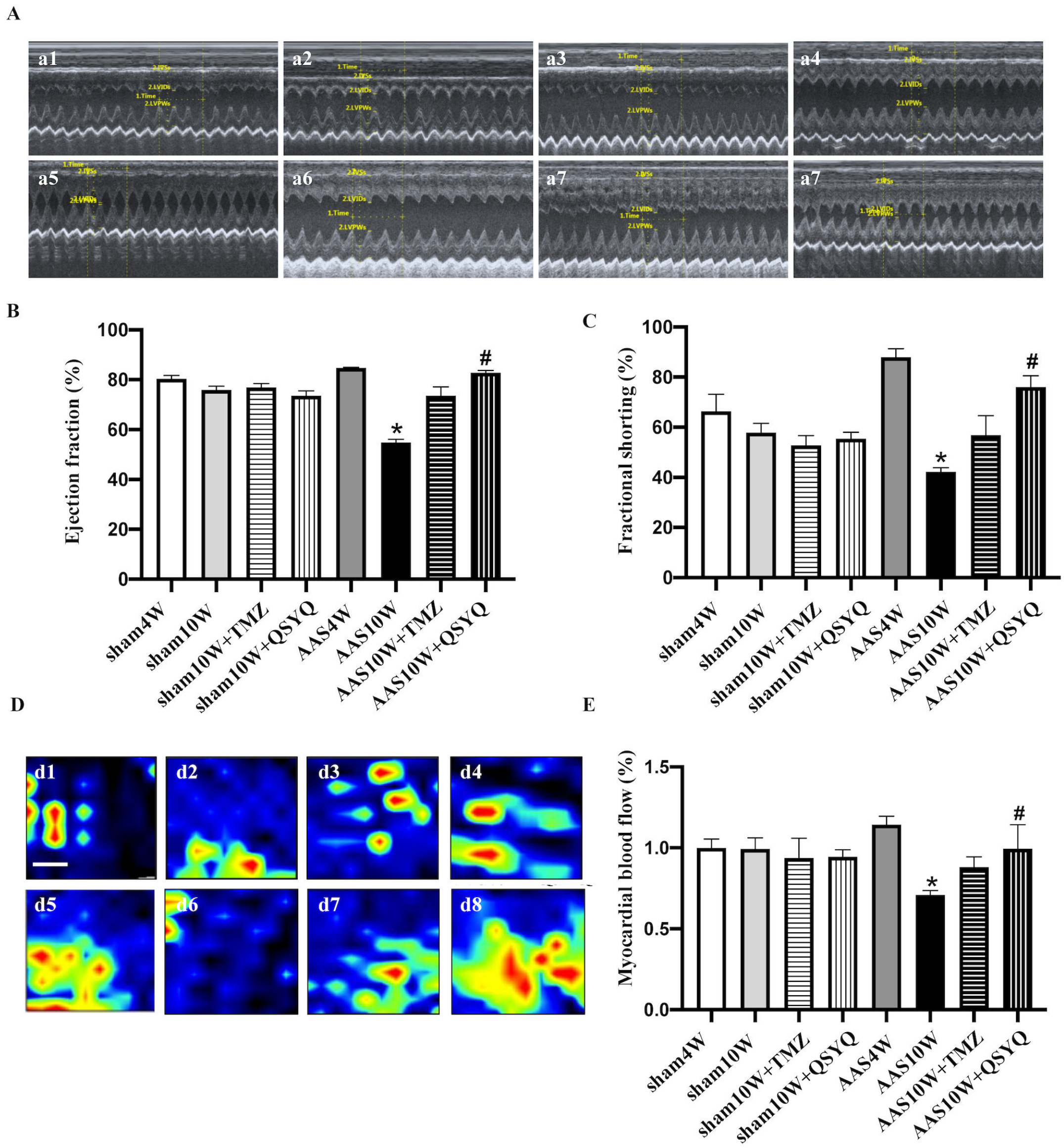

Supplementary Fig. 1. QSYQ post-treatment improves left ventricular (LV) contractility, chamber remodeling and myocardial blood flow in AAS rats. The LV parameters were assessed based on an echocardiography analysis at 4 weeks and 10 weeks after AAS surgery, respectively. (**A**) Representative images showing the M mode echocardiography of rats in the Sham4W (a1), Sham10W (a2), Sham10W+TMZ (a3), Sham10W+QSYQ (a4), AAS4W (a5), AAS10W (a6), AAS10W+TMZ (a7), and

AAS10W+QSYQ (a8) groups. (**B**) Ejection fraction (EF%) in the different groups. (**C**) Fractional shortening (FS%) in the different groups. Data are presented as the mean ± SEM, n = 6. (**D**) Representative color images of heart acquired by Laser-Doppler Perfusion Imager in in Sham4W (d1), Sham10W (d2), Sham10W+TMZ (d3), Sham10W+QSYQ (d4), AAS4W (d5), AAS10W (d6), AAS10W+TMZ (d7), AAS10W+QSYQ (d8) groups; (**E**) Quantitative evaluation of the MBF. Data are presented as the mean ± SEM, n = 6. * $P < 0.05$ *vs.* sham; $^{\#}$ $P < 0.05$ *vs.* AAS.

Supplementary Figure 2

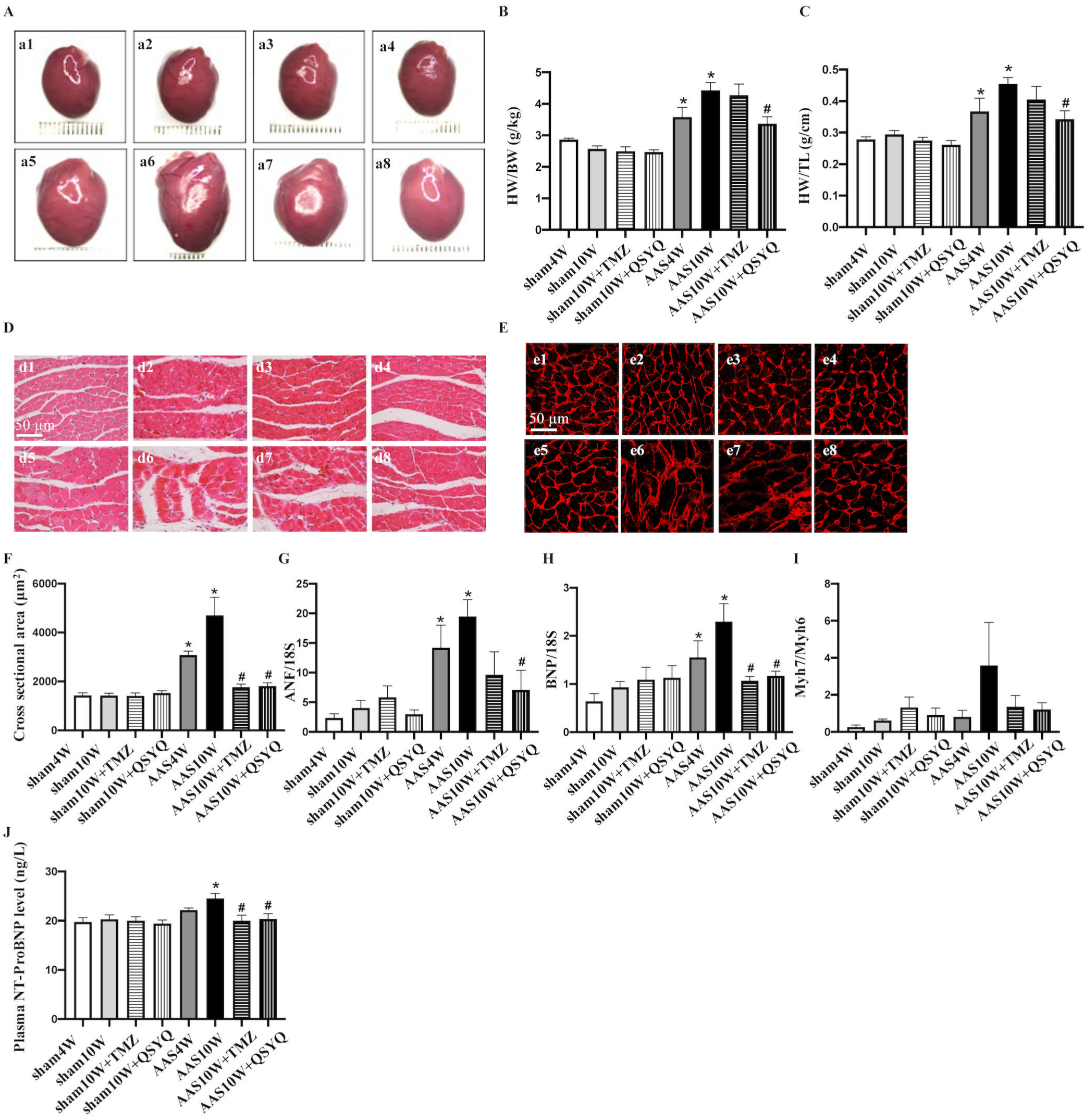

Supplementary Fig. 2. QSYQ post-treatment reduced pressure overload-induced

cardiac hypertrophy in rat. (**A**) Representative photographs showing the macroscopic features of cardiac hypertrophy induced by AAS challenge in the Sham4W (a1), Sham10W (a2), Sham10W+TMZ (a3), Sham10W+QSYQ (a4), AAS4W (a5), AAS10W (a6), AAS10W+TMZ (a7), and AAS10W+QSYQ (a8) groups. (**B and C**) Quantitative evaluation of HW/BW and HW/TL. Data are presented as the mean ± SEM, n = 6. (**D**) Representative images of HE and WGA staining (**E**) of myocardium in the Sham4W (d1, e1), Sham10W (d2, e2), Sham10W+TMZ (d3, e3), Sham10W+QSYQ (d4, e4), AAS4W (d5, e5), AAS10W (d6, e6), AAS10W+TMZ (d7, e7), and AAS10W+QSYQ (d8, e8) groups. Scale bar = 50 μm. (**F**) Quantification of WGA staining showing the cross-sectional area. (**G-I**) ANF/18S, BNP/18S and Myh7/Myh6 in the different groups. (**J**) NT-ProBNP level in the plasma of AAS-induced rat was detected by ELISA. Data are-presented as the mean ± SEM, n = 3. * $P < 0.05$ *vs.* sham; $^{\#}$ $P < 0.05$ *vs.* AAS.

Supplementary Figure 3

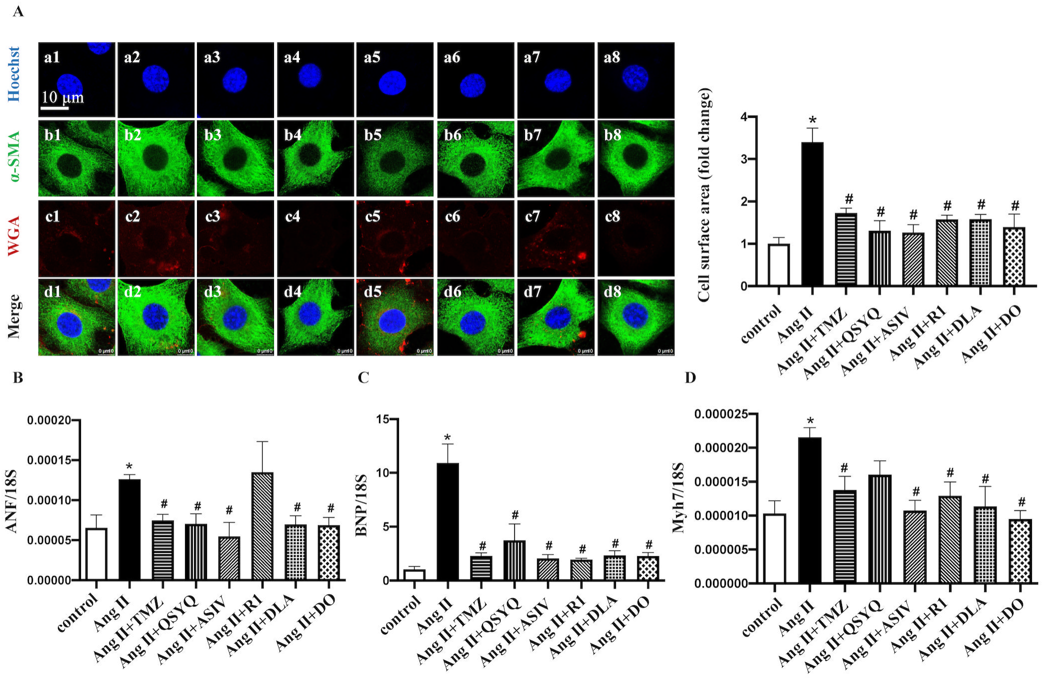

Supplementary Fig. 3. Effects of QSYQ and its main ingredients on Ang II induced H9C2 cardiomyocyte hypertrophy. (**A**) Immunofluorescence images of α-SMA (green), WGA (red), and nuclei (blue) in H9C2 cells in the control (a1, b1, c1, d1), Ang II (a2, b2, c2, d2), Ang II+TMZ (a3, b3, c3, d3), Ang II+QSYQ (a4, b4, c4, d4), Ang II+ASIV (a5, b5, c5, d5), Ang II+R1 (a6, b6, c6, d6), Ang II+DLA (a7, b7, c7, d7), and Ang II+DO (a8, b8, c8, d8) groups and quantification results of the cell surface area. Data are presented as the mean ± SEM, n = 3. Scale bar = 10 μm. (**B-D**) ANF, BNP, and Myh7 mRNA levels in the different groups. Data are presented as the mean ± SEM, n = 4. * $P < 0.05$ *vs.* control; $^{\#}$ $P < 0.05$ *vs.* Ang II.

Supplementary Figure 4

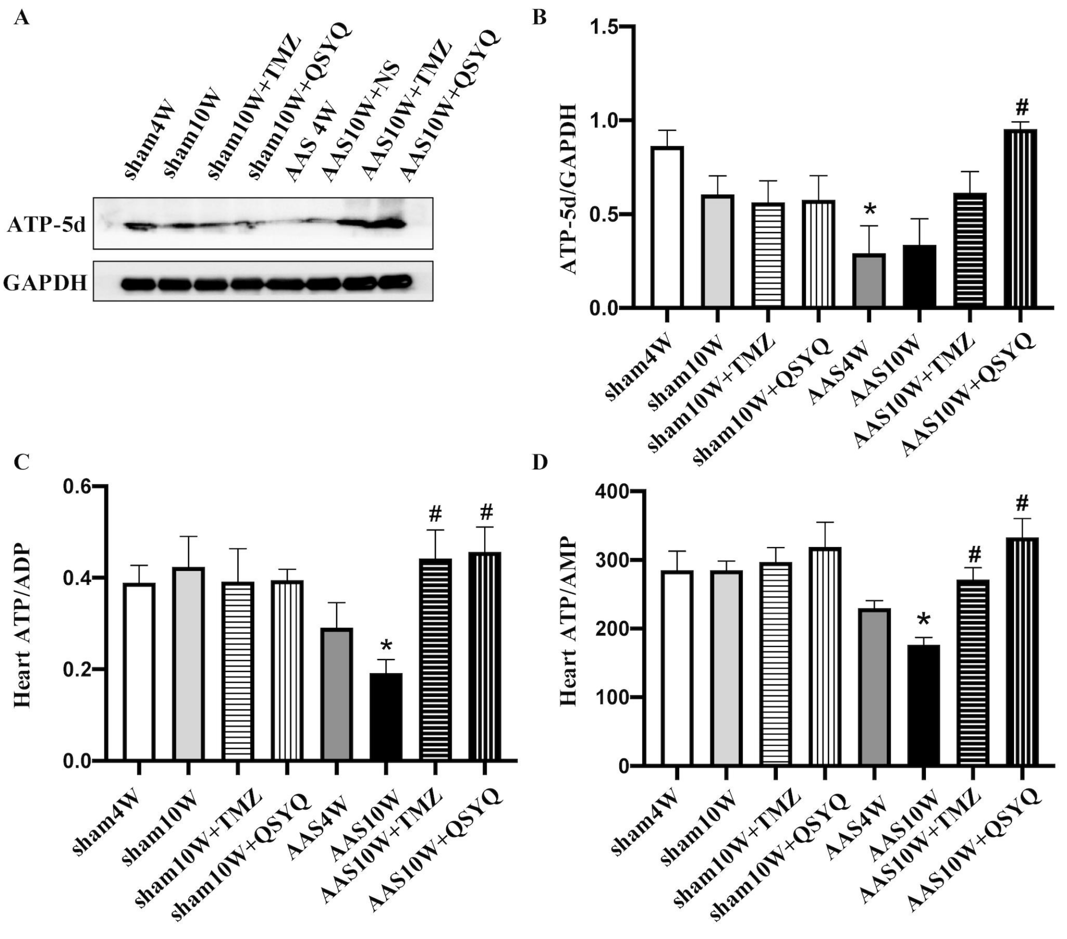

Supplementary Fig. 4. QSYQ post-treatment ameliorated pressure overload-induced cardiac energy metabolism disorder in rat. (**A**) Expression of ATP-5d in rat myocardium examined by western blot for the different groups. (**B**) Quantitative evaluation of the western blot results of ATP-5d. (**C**) Representative ATP/ADP ratio in rat myocardium in the different groups. (**D**) Representative ATP/AMP ratio in rat myocardium in the different groups. Data are presented as the mean ± SEM, n = 4. * $P < 0.05$ *vs.* sham; $^{\#}$ $P < 0.05$ *vs.* AAS.

Supplementary Figure 5

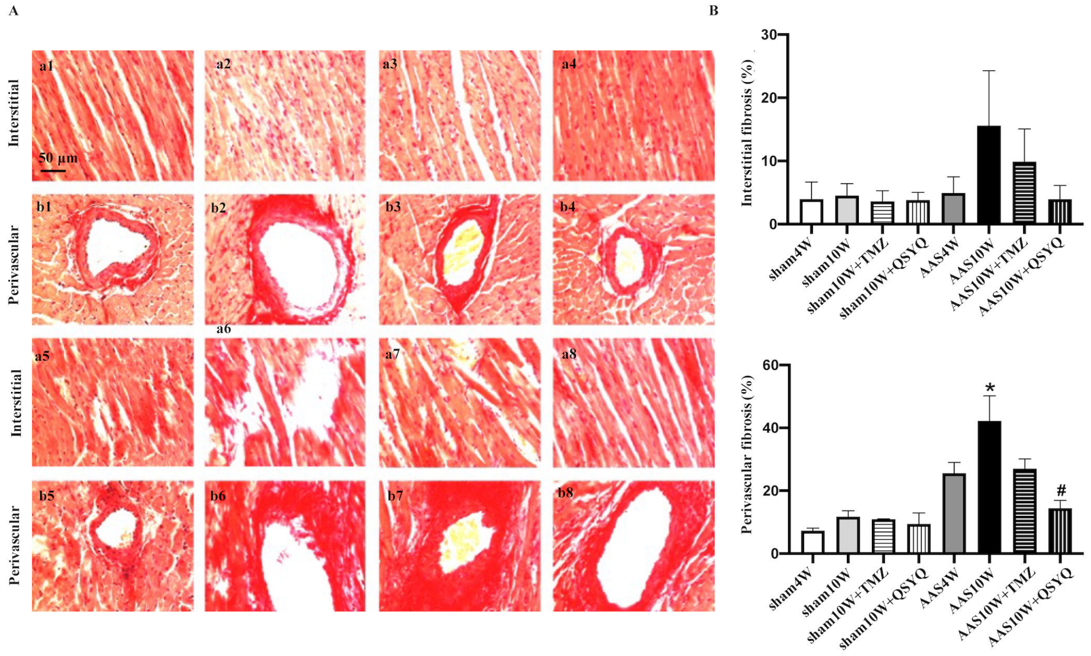

Supplementary Fig. 5. Effects of QSYQ on collagen deposition in AAS-induced rat myocardium. (**A**) Sirius red staining of collagen in rat myocardium in the Sham4W (a1, b1), Sham10W (a2, b2), Sham10W+TMZ (a3, b3), Sham10W+QSYQ (a4, b4), AAS4W (a5, b5), AAS10W (a6, b6), AAS10W+TMZ (a7, b7), AAS10W+QSYQ (a8, b8) groups. Scale bar = 50 μm. (**B**) Quantification results of intestinal and perivascular fibrosis determined by Sirius Red staining. Data are presented as the mean ± SEM, n = 3. * $P < 0.05$ *vs.* sham; # $P < 0.05$ *vs.* AAS.

Supplementary Figure 6

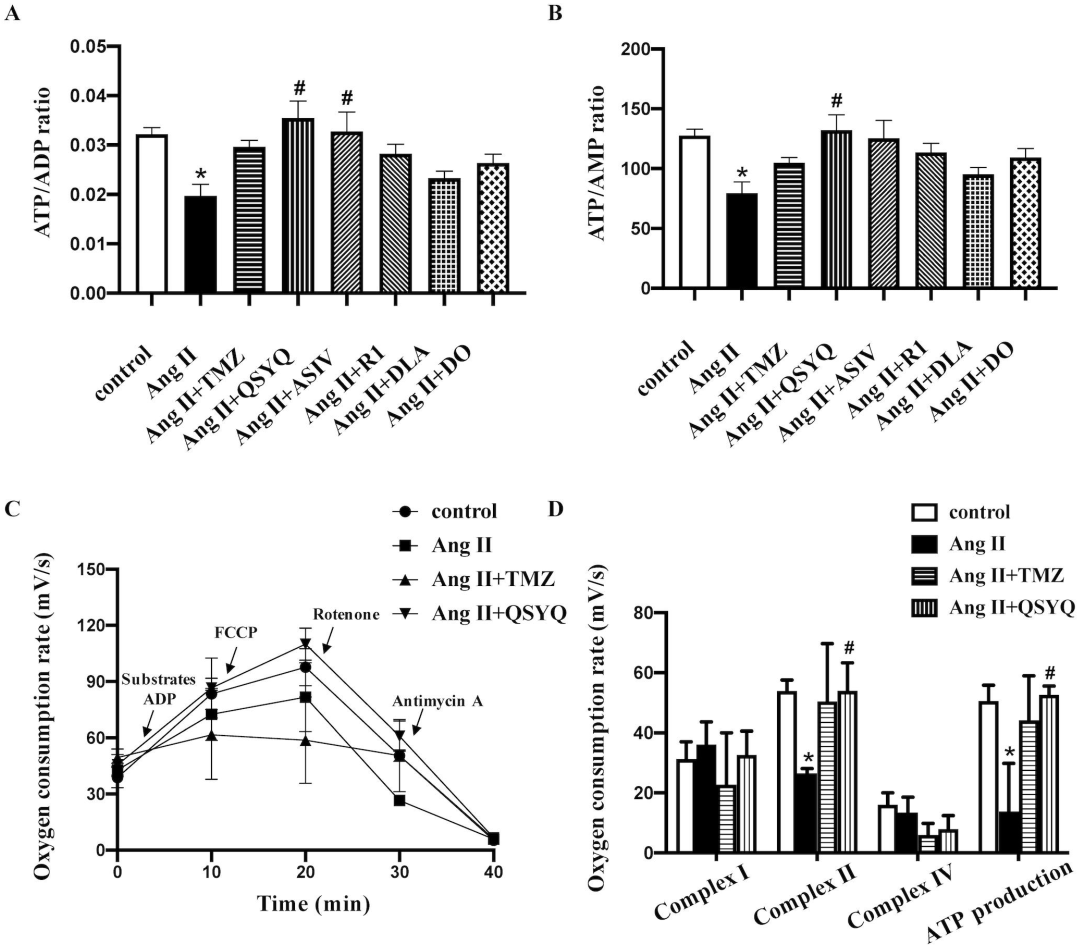

Supplementary Fig. 6. Effects of QSYQ and its main ingredients on Ang II-induced H9C2 cell energy metabolism disorder. (**A**) ATP/ADP ratio in H9C2 cells from the different groups. (**B**) ATP/AMP ratio in H9C2 cells from the different groups. (**C**) Average OCR showing respiratory activity measured by Oxyraph-2k. Arrows indicate the sequential additions of mitochondrial substrates to assess the respiratory states in H9C2 cells. (**D**) Mitochondrial complex activities measured by Oxyraph-2k in H9C2 cells. Data are presented as the mean ± SEM, n = 4. * $P < 0.05$ *vs.* control; $^{\#}$ $P < 0.05$ *vs.* Ang II.

Supplementary Figure 7

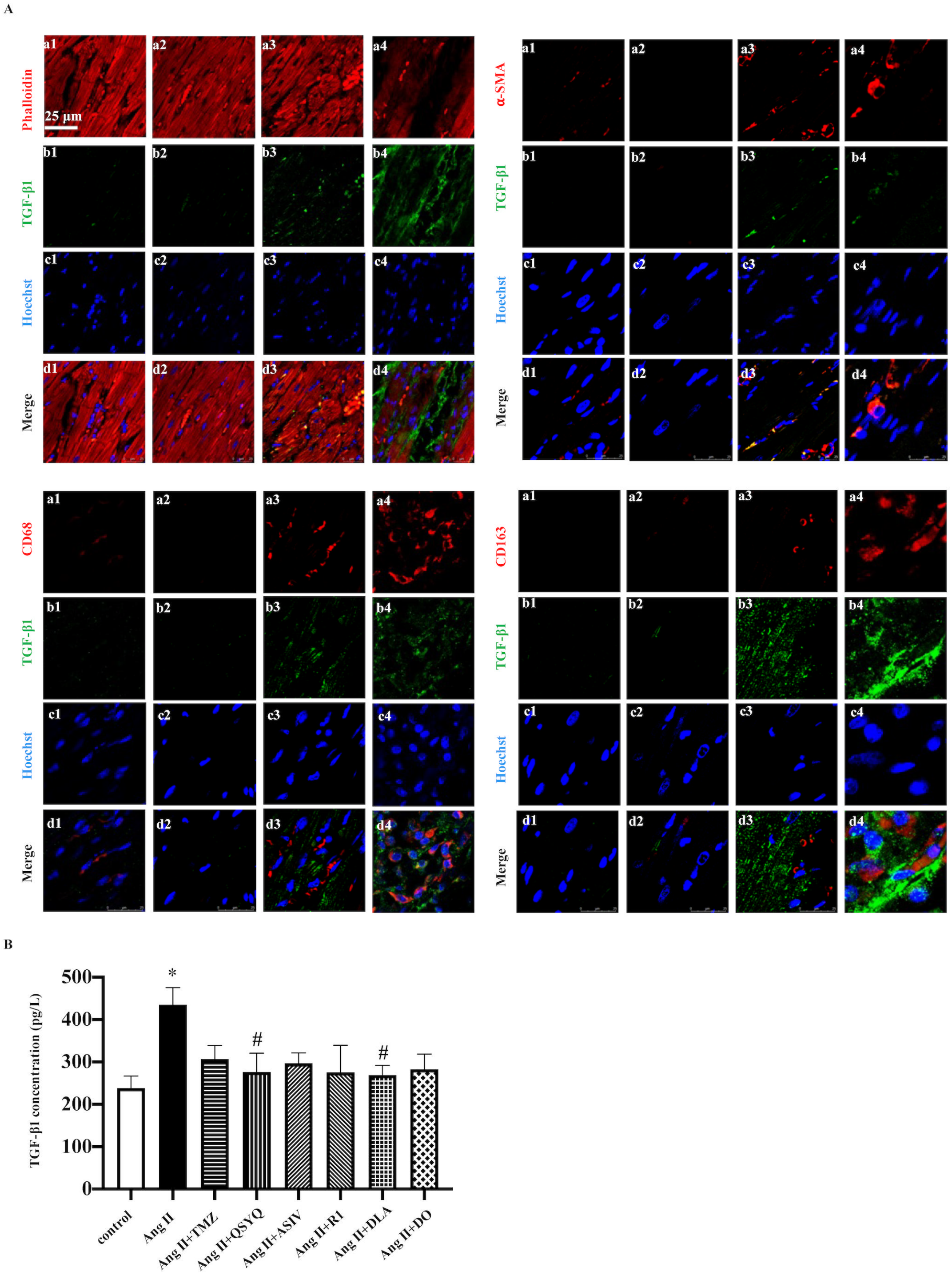

Supplementary Fig. 7. TGF-β 1 content in AAS-challenged rat myocardium and Ang II-induced H9C2 cardiac myocytes. (**A**) Immunofluorescence images of TGF-β1, phalloidin, α-SMA, CD68, CD163 and nuclei in rat myocardium from the Sham4W

(a1, b1, c1, d1), Sham10W (a2, b2, c2, d2), AAS4W (a3, b3, c3, d3), and AAS10W (a4, b4, c4, d4) groups. Scale bar = 25 μm. (**B**) TGF-β1 expression level in the supernatants of H9C2 cells detected by ELISA. Data are presented as the mean ± SEM, n = 4. * $P < 0.05$ *vs.* control; $^{\#}$ $P < 0.05$ *vs.* Ang II.

Supplementary Figure 8

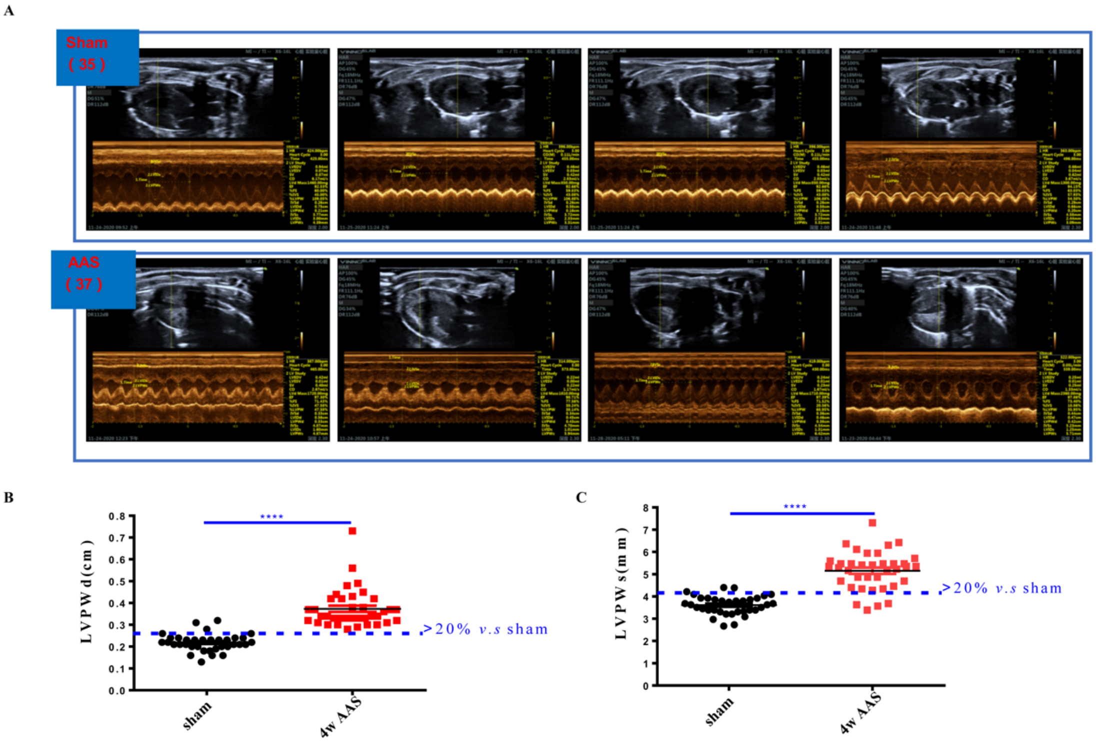

Supplementary Fig. 8. LV parameters were assessed based on an echocardiography analysis 4 weeks after AAS challenge. (**A**) Representative images showing the M mode echocardiography of rat. (**B**) Relative end diastolic left ventricular posterior wall thickness (LVPWd). (**C**) Relative end systolic left ventricular posterior wall thickness (LVPWs). Data are presented as the mean ± SEM, n = 35. *** $P < 0.001$ *vs.* sham.

Supplementary Figure 9

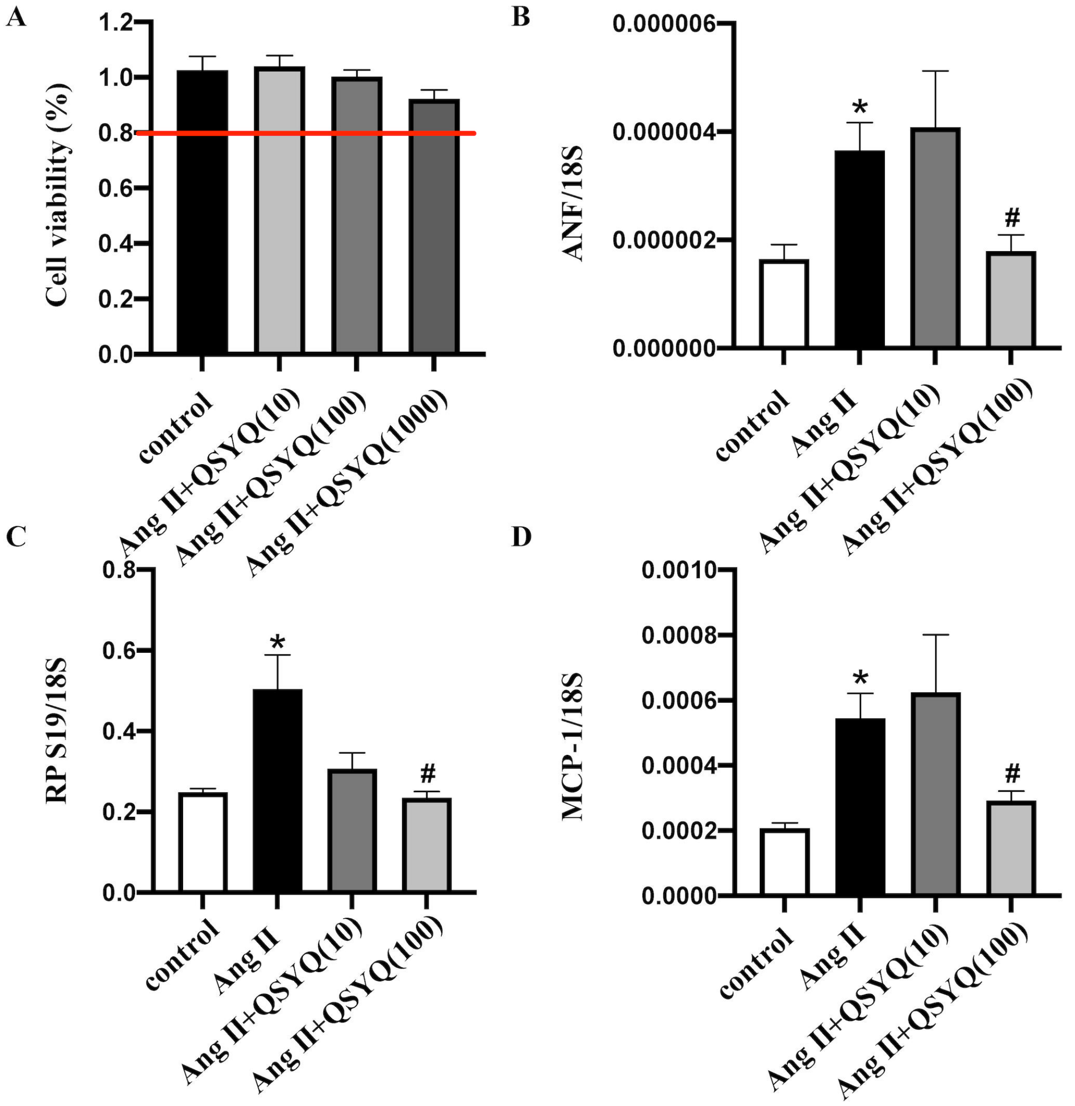

Supplementary Fig. 9. Effects of different dosage of QSYQ on Ang II-induced H9C2 cell hypertrophy and release of chemokine RP S19 and MCP-1. (**A**) CCK-8 assay result of different dosage of QSYQ in Ang II cell stimulated H9C2 cells. (**B-D**) ANF, RP S19, and MCP-1 mRNA levels in the different groups in H9C2 cells. Data are presented as the mean ± SEM, n = 3. * $P < 0.05$ *vs.* control; $^{\#}$ $P < 0.05$ *vs.* Ang II.
